# Designing of banana shaped chromophores via molecular engineering of terminal groups to probe photovoltaic behavior of organic solar cell materials

**DOI:** 10.1038/s41598-023-39496-6

**Published:** 2023-09-12

**Authors:** Saeed Ahmed, Iram Irshad, Saima Nazir, Salma Naz, Muhammad Adnan Asghar, Saad M. Alshehri, Saifullah Bullo, Muhammed Lamin Sanyang

**Affiliations:** 1 Department of Pharmaceutical Sciences, University of Milan, Via Venezian 21, 20133 Milan, Italy; 2 Institute of Chemistry, Khwaja Fareed University of Engineering and Information Technology, Rahim Yar Khan, 64200 Pakistan; 3 Centre for Theoretical and Computational Research, Khwaja Fareed University of Engineering and Information Technology, Rahim Yar Khan, 64200 Pakistan; 4 Nawaz Sharif Medical College, University of Gujrat, Gujrat, Pakistan; 5 Institute of Biological Sciences, Khwaja Fareed University of Engineering and Information Technology, Rahim Yar Khan, 64200 Pakistan; 6 Department of Chemistry, Division of Science and Technology, University of Education Lahore, Lahore, Pakistan; 7 Department of Chemistry, College of Science, King Saud University, Riyadh, Saudi Arabia; 8 Department of Human and Rehabilitation Sciences, Begum Nusrat Bhutto Women University, Sukkur Sindh, Pakistan; 9 Directorate of Research and Consultancy, University of The Gambia, Kanifing Campus, MDI Road, P.O Box 3530, Serekunda, The Gambia

**Keywords:** Chemistry, Materials science, Optics and photonics

## Abstract

To meet the rising requirement of photovoltaic compounds for modernized hi-tech purpose, we designed six new molecules (DTPD1-DTPD6) from banana shaped small fullerene free chromophore (DTPR) by structural tailoring at terminal acceptors. Frontier molecular orbitals (FMOs), density of states (DOS), open circuit voltage (*V*_oc_), transition density matrix (TDM) analysis, optical properties, reorganization energy value of hole and electron were determined utilizing density function theory (DFT) and time-dependent density function theory (TD-DFT) approaches, to analyze photovoltaic properties of said compounds. Band gap contraction (∆E = 2.717–2.167 eV) accompanied by larger bathochromic shift (*λ*_max_ = 585.490–709.693 nm) was observed in derivatives contrary to DTPR. The FMOs, DOS and TDMs investigations explored that central acceptor moiety played significant role for charge transformation. The minimum binding energy values for DTPD1-DTPD6 demonstrated the higher exciton dissociation rate with greater charge transferal rate than DTPR, which was further endorsed by TDM and DOS analyses. A comparable *V*_oc_ (1.49–2.535* V*) with respect to the HOMO_PBDBT_–LUMO_acceptor_ for entitled compounds was investigated. In a nutshell, all the tailored chromophores can be considered as highly efficient compounds for promising OSCs with a good *V*_*oc*_ response.

## Introduction

Solar power technology based devices convert the light energy into the electricity under known photovoltaic effect^1^. Silicon-built materials offer an exceptional power conversion efficiency (PCE) of 46%, however their application in organic solar cells is restricted because of steep cost, complex manufacturing, static energy orbitals besides structural constraints^2,3^. Furthermore, thin-film organic-based solar cell devices offer benefits like low-temperature manufacturing, insubstantial design and cost-effectiveness, but their drawbacks such as environmental pollution, high price and lack of structural tunability make them less favorable^4–6^. Recent studies have drawn attention to metal-free organic sensitizers because of their structural adjustability, high molar extinction coefficient, cost-effectiveness as well as eco-friendly nature, making them favorable for use in organic solar cell applications^4,7^. Fullerene-based derivatives have intrinsic deficiencies that can be addressed by fullerene-free small molecule acceptors (NFSMAs). Due to the changeable energy levels, high morphological reliability, broad optical absorption and cost-effective production NFSMAs has become suitable candidate over typical fullerene-based acceptors ^8,9^. Significant research efforts are being made on NFSMAs because they are excellent candidates for extremely effective OSCs. Acceptor–donor-acceptor (A-D-A) architecture is among the mainly fruitful architectures of NFA-built OSCs, among all the classified NFA type structures^10^. Amongst numerous kinds of small moieties comprising donor–acceptor (D-A) architecture, the A-*π*-A-*π*-A type, with its electron-accepting central core, is a potential acceptor molecule for bulk heterojunction OSCs due to its low-lying HOMO including high open-circuit voltage (*V*_oc_) ^11–17^. Chen’s et al*.,* yielded a small molecule DR_3_TSBDT having dialkyl-thiol substituted BDT acting central unit and 3-ethylrhodanine acting terminal segment which revealed an elevated PCE of 9.95%^18,19^. Wei’s et al., studied a set of small moieties containing thiophene-substituted benzodithiophene acting central and fluorinated 1*H*-indene-1,3(2*H*)-dione acting the terminal moieties, amongst which a high PCE of 11.08% was attained. Qian’s et al*.,* drafted and synthesized acceptor2-*π*-acceptor1-*π*-acceptor2 type small acceptor moiety which incorporated a strong electron-accepting moiety, 3-bis(4-(2-ethylhexyl)-thiophen-2-yl)-5,7-bis(2ethylhexyl)benzo[1,2-:4,5-*c*’]-dithio phene-4,8-dione as the core electron accepting moiety, thiophene-alkoxy benzene-thiophene as the pi-bridge, and indenedione as terminal group moiety^20–23^. Thus, taking the clue from enhancement in PCE by end-group redistribution, we drafted six new acceptor type molecules with A2-π-A1-π-A2 type architecture (DTPR-DTPD6), and their optoelectronic assets are estimated to be utilized as electron donating materials in OSCs. The electronic, photophysical and photovoltaic properties of DTPR-DTPD6 are assessed and efficient electron acceptor molecules are tailored for their application in OSCs.

### Computational detail

**Figure 1:**
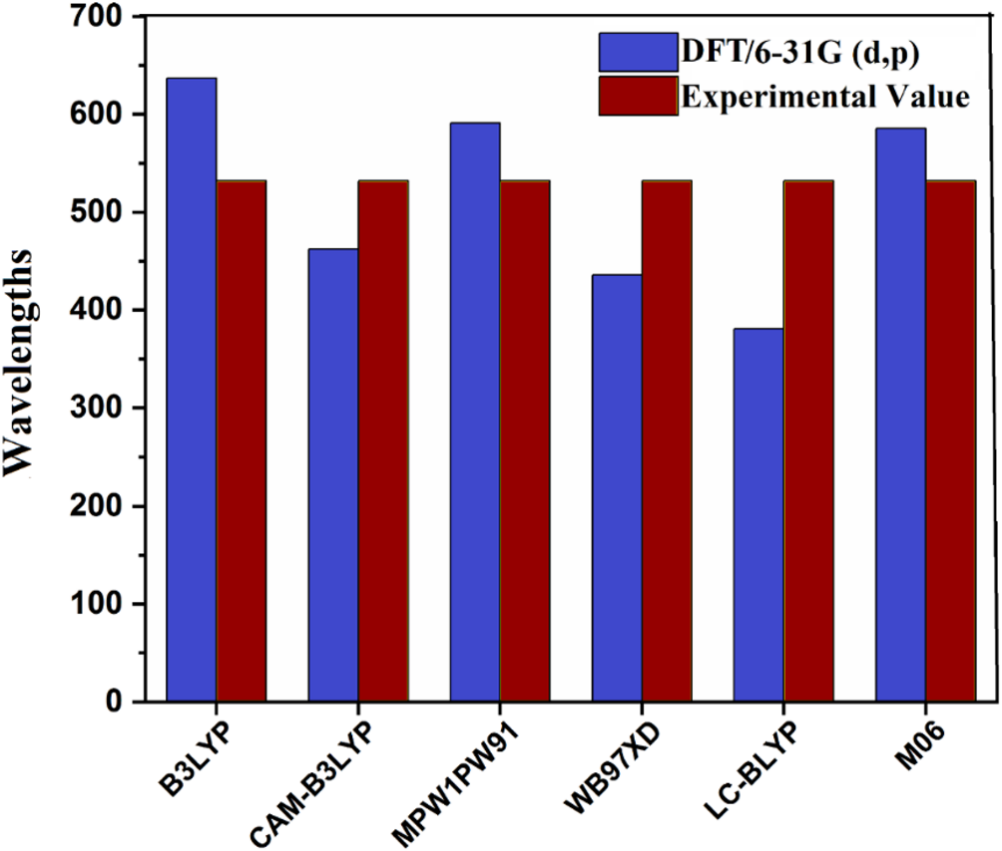
Comparison between DFT and experimental *λ*_max_ for the selection of functional.

Gaussian 09 package^24^ was employed to conduct all quantum chemical calculations for current study. With the aid of GaussView 6.0 package^25^, inputs for BDD-IN and its DTPD1-DTPD6 were developed. The selection of sophisticated functional was done through benchmarked against experimental results (maximum absorption). For this purpose, DTPR chromophore was optimized at M06 functional of DFT^26^, ^27^ alongside 6–31G(d,p)^28^ basis set to obtain true minima structures. Then these optimized geometries were utilized to perform UV–Vis analysis in chloroform solvent. The *λ*_max_ outcomes of DTPR examined in chloroform at foresaid functional were 636.82, 462.21, 591.16, 435.64, 380.91, and 585.49 nm, and the experimentally determined *λ*_max_ of DTPR was reported to be 532 *nm*^29^ at M06 functional, closed harmony was seen with experimental value as shown in Fig. 1 hence, this functional was selected for this study. All the derivatives of DTPR were optimized at M06/6-31G(d,p) for acquiring ground state geometries. After the successful optimization of geometries a wide array of investigations including reorganization energy (RE), frontier molecular orbitals (FMOs), open circuit voltage (*V*_oc_), density of states (DOS), binding energy (*E*_b_), transition density matrix (TDM) and charge transference analyses were accomplished to explore the photovoltaic properties of afore-said chromophores. The RE was calculated by utilizing the Equations S1 and S2. Different softwares: PyMOlyze 1.1^30^, Multiwfn 3.7^31^, Origin 6.0^32^, Avogadro^33^ and Chemcraft^34^ were utilized to interpret results from the output files in the forms of tabular and pictorial data.

## Results and discussion

**Figure 2:**
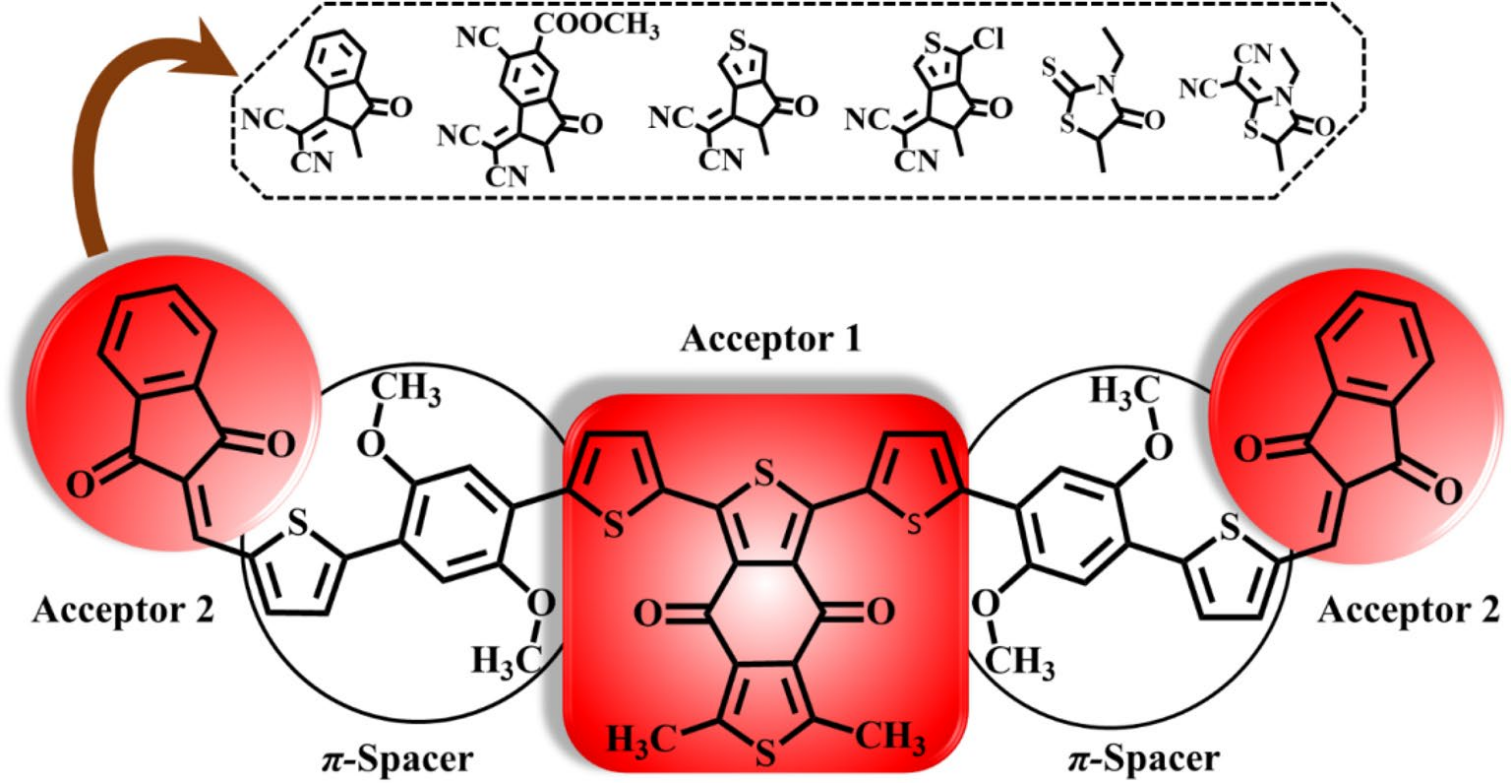
The schematic representation of DTPR and derivatives.

For current investigation, dimethyl benzodithiophene-4,8-dione based organic (BDD-IN)^22^ system with A_2_-*π*-A_1_-*π*-A_2_ configuration is selected which is synthesized by Zhang et al*.* This BDD-IN chromophore consists of three parts 1) core: alkoxy benzodithiophene-4,8-dione (BDD) which is acting as central acceptor (A1) 2) indenedione (IND) end-cap electron accepting moieties (A2) and 3) π-spacer: 2,2'-(2,5-dimethoxy-1,4-phenylene)dithiophene that connects the terminal acceptors (A2) with core acceptor (A1). With the aim of minimizing computational cost and time, the large alkyl chain in BDD-IN is replaced with methyl group and named as DTPR. Further, molecular engineering of this DTPR chromophore at terminal units (A2) is done with various efficient acceptors (Fig. 2) and effect of these acceptors on the photovoltaic properties is explored through DFT.

**Figure 3:**
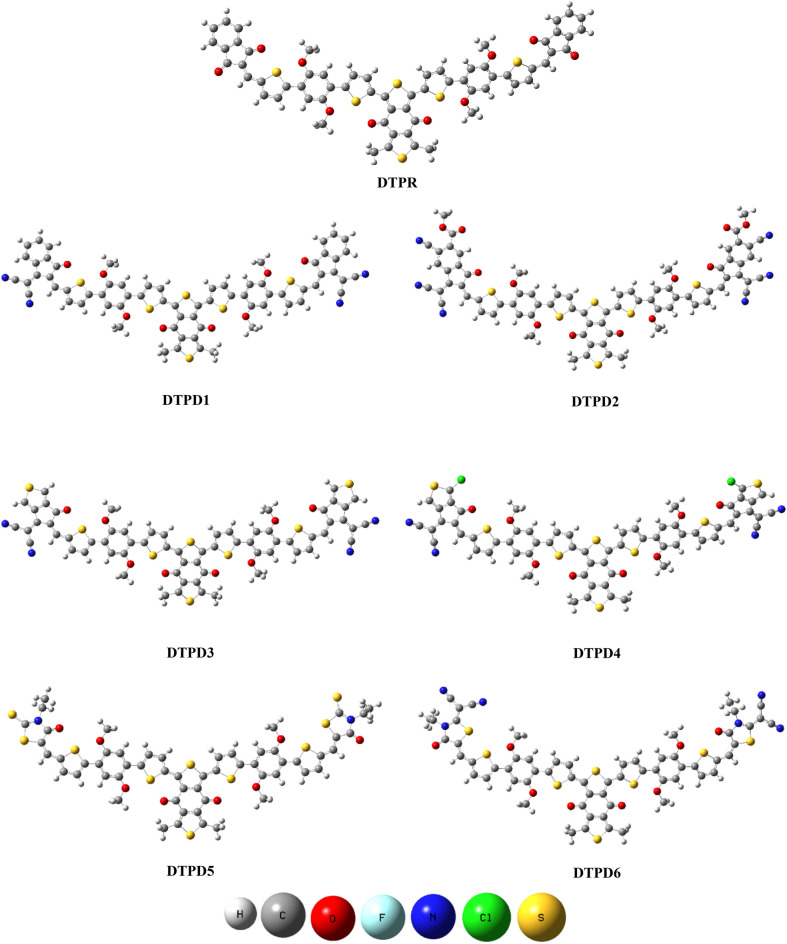
Optimized structures of DTPR and DTPD1-DTPD6. Pictures are created by GaussView 5.0 and Gaussian 09 version D.01 (https://gaussian.com/g09citation/).

The IUPAC names of structures and various acceptors used for structural tailoring are displayed in supplementary data (Tables S11–S12). The ground state optimized structures present at true minima in potential energy surfaces are illustrated in Fig. 3 whereas, chemical structures and Cartesian coordinates are shown in Fig. S1 and Tables S1–S7, respectively.

### Frontier molecular orbitals (FMOs)

Frontier molecular orbitals (FMOs) propagation pattern is an outstanding technique for the description of electronic and optical properties^35^. Energy difference (*E*_g_ = *E*_LUMO_ − *E*_HOMO_) is regarded as an essential characteristic that gives insight on the photovoltaic efficiency of photovoltaic materials^36^. As per band theory, lowest unoccupied molecular orbitals (LUMO) and highest occupied molecular orbitals (HOMO) are described by means of valence and conduction band, respectively^37^. DFT is used to estimate the energy values of HOMO, LUMO as well as *E*_g_ of investigated chromophores (DTPR and DTPD1-DTPD6) and outcomes are recorded in Table 1. The intramolecular charge transfer (ICT) is boosted through conjugation and delocalization of the electrons within the molecular systems. The *E*_g_ serves as a predictor for, catalyst behind open circuit voltage (*V*_oc_) and exciton dissociation. Lower the energy difference, easier is the charge transfer which simultaneously increases the PCE of OSCs. It also illustrates the dynamic strength, electron transmission properties, chemical hardness, softness and excitability of DTPR and DTPD1-DTPD6^38^.

**Table 1:** FMOs energies and their difference (*E*_*g*_) of DTPR and DTPD1-DTPD6 at aforesaid level of DFT and basis set.

Compounds	*E*_HOMO_	*E*_LUMO_	*E*_g_
DTPR	− 5.275	− 2.558	2.717
DTPD1	− 5.450	− 3.009	2.441
DTPD2	− 5.778	− 3.611	2.167
DTPD3	− 5.482	− 3.074	2.408
DTPD4	− 5.452	− 2.818	2.634
DTPD5	− 5.272	− 2.566	2.706
DTPD6	− 5.452	− 2.818	2.634

*E*_HOMO_ = Energy of HOMO, *E*_LUMO_ = Energy of LUMO, *E*_g_ = *E*_LUMO_ − *E*_HOMO_.

**Figure 4:**
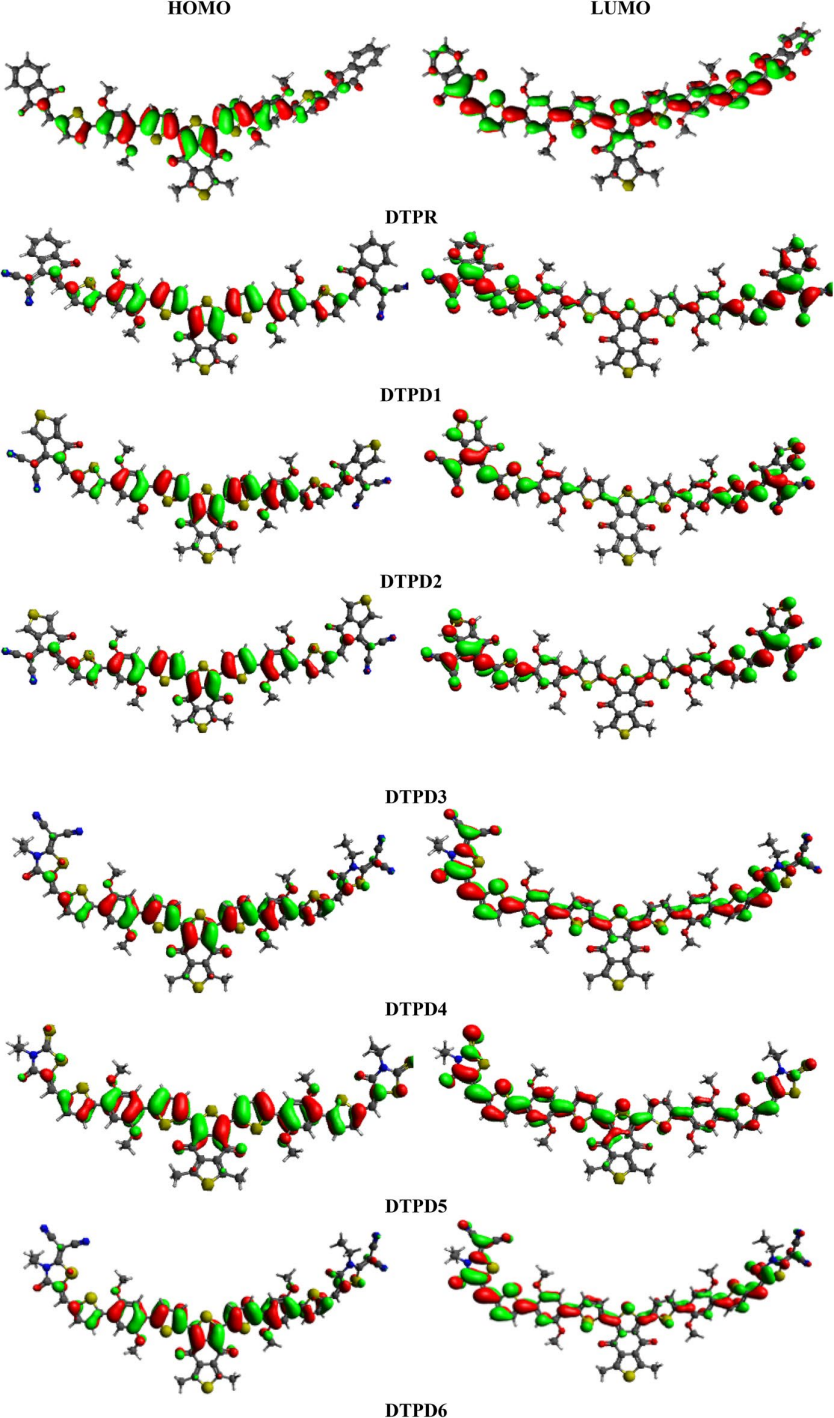
FMOs graphic depiction for DTPR and DTPD1-DTPD6 units are in *eV.* Illustrations are made using Avogadro software, Version 1.2.0. (http://avogadro.cc/).

Computed *E*_HOMO_ values for DTPR and DTPD1-DTPD6 are − 5.275, − 5.450, − 5.778, − 5.482, − 5.452, − 5.272 and − 5.452 eV, respectively. While, *E*_LUMO_ values of DTPR and DTPD1-DTPD6 are − 2.558, − 3.009, − 3.661, − 3.074, − 2.818, − 2.566 and − 2.818 eV, respectively. Similarly, the *E*_g_ values for DTPR and DTPD1-DTPD6 are found to be 2.717, 2.441, 2.167, 2.408, 2.634, 2.706 and 2.634 eV, respectively. Among all investigated compounds, DTPD5 possessing EMT as terminal acceptor depicted lower *E*_g_ (2.706 eV) than the reference compound (2.717 eV) owing to the sulphur atom at peripheral acceptors which lessen the *E*_g_ between HOMO/LUMO orbitals. This *E*_g_ is further reduced to 2.634 eV in DTPD6 and DTPD4 due to the incorporation of cyano and chloro groups at the groups at the structure of EMI and CTM (terminal acceptors), respectively possessing improved resonance and electron withdrawing nature. The greater negative inductive effect of cyano and chloro possibly the cause of this reduction in band gap. The *E*_g_ is observed to be declined to 2.441 eV, manifested by DTPD1, owing to the MIM acceptor arm. A further drop in *E*_g_ is noticed in DTPD3, which has MTM as acceptor motif that is largely effective in improving the resonance. The lowermost value of *E*_g_ (2.167 eV) is observed in DTPD2 which has acetyl group on the terminal acceptor motif MMC. In short, the *E*_g_ of all the designed chromophores decline in the following order as: DTPR > DTPD5 > DTPD6 = DTPD4 > DTPD1 > DTPD3 > DTPD2. Furthermore, the FMO diagrams has been used to explicit the charge transference phenomenon, as represented in Fig. 4.

The HOMO charge concentration is circulated around the central electron accepting unit and a small quantity of charge is detected on *π*-linker portion, while LUMO is distributed principally on terminal electron accepting moieties of DTPR, DTPD1, DTPD2 and DTPD3 as represented in Fig. 4. For DTPD4, DTPD5 and DTPD6, the charge density of HOMO is dispersed on central electron accepting units and a small quantity of charge is noticed at $\pi$-linker portion while, LUMO is majorly distributed across the acceptor moieties, moderately over the *π*-linker and minutely on the central acceptor moieties. These charge density rotations illustrate the successful electrons transmission and a maximum electron transfer from D to A region with the support of *π*-spacer in all the investigated compounds. Moreover, the values of HOMO-1/LUMO + 1 and HOMO-2/LUMO + 2 are presented in Table S8.

### Density of states (DOS)

**Figure 5:**
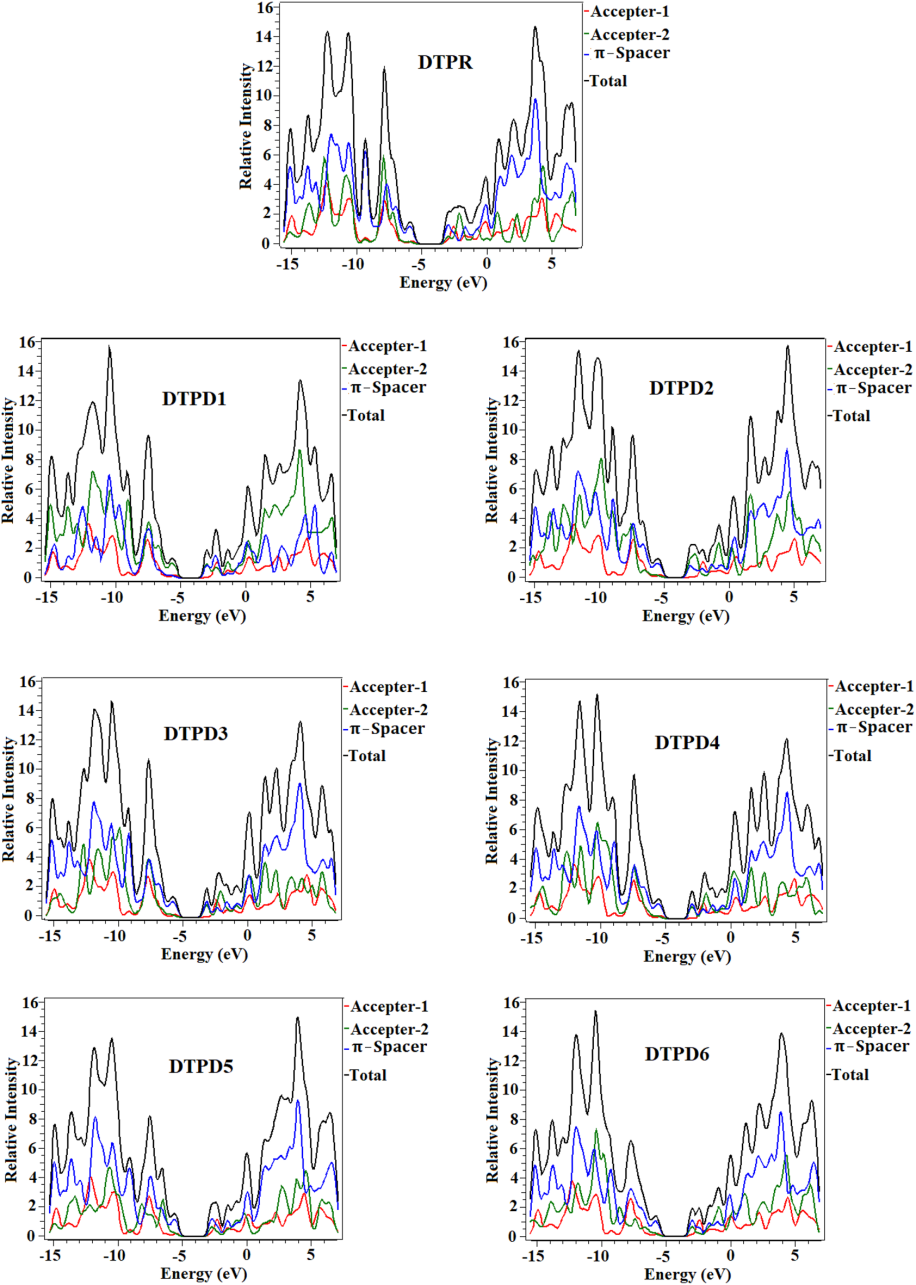
Pictorial illustration of DOS of investigated molecules (DTPR and DTPD1-DTPD6). The DOS pictographs are drawn utilizing PyMOlyze 1.1 version.

To confirm the conclusions drawn from FMOs analysis, DOS is accomplished using the M06/6-31G(d,p) method, and the PyMOlyze 1.1^30^ suite is utilized to obtain a curve plot^39^. The pattern of electronic charge dissemination on FMOs could be altered by the using various terminal acceptors which could be further validated by the DOS percentages of HOMO–LUMO. ^40,41^ Fig. 5 demonstrates that the distribution of electronic charge around the HOMO–LUMO which is influenced by changing the acceptors at the terminal position. The reference (DTPR) and designed chromophores (DTPD1-DTPD6) are partitioned into three fragments namely as acceptor 1, acceptor 2 and *π*-linker, arranged as A2-*π*-A1-*π*-A2. Figure 5 revealed that highest peak for charge dissemination for HOMO is observed at *π*-spacer at -12 eV; in case of LUMO it appears at acceptor 2 at 4 eV in all the studied compounds. Further, DOS analysis reveals a noteworthy distribution and delocalization of electron. Additionally, a substantial charge transfer is observed, with a significant magnitude of charge being transmitted out of the central acceptor to the end-capped acceptor. The contribution of electrons from each individual fragment is visually depicted by separate bands of different colors. In contrast, the black-colored band signifies the collective electronic contribution encompassing the entire molecule. The red, green and blue indicate charge concentration in the HOMO and LUMO of acceptor 1, acceptor 2 and *π*-linker, respectively, as shown in Fig. 5. Acceptor 1 (BT) depicts charge distribution pattern as 13.4, 3.6, 1.8, 3.4, 2.9, 12.6 and 7.4% to HOMO and 19.3, 20.4, 21.2, 20.4, 20.5, 18.4 and 91.3% to LUMO in DTPR and DTPD1-DTPD6, respectively. Acceptor 2 contributes 22.3, 52.5, 56.5, 41.0, 42.1 28.1 and 32.7% to HOMO and 6.5, 72.2, 7.4, 7.7, 7.7, 9.7 and 8.7% to LUMO in DTPR and DTPD1-DTPD6, respectively. Similarly, *π*-linker contributes 64.2, 44.0, 41.7, 55.5, 55.0, 59.2 and 59.8% to HOMO and 74.2, 7.4, 71.3, 71.9, 71.8, 71.9 and 72.1% to LUMO in DTPR and DTPD1-DTPD6, correspondingly. These contributions affirm that different types of electronic transmission can be accomplished via altering peripheral acceptor motifs.

### Optical properties

**Figure 6:**
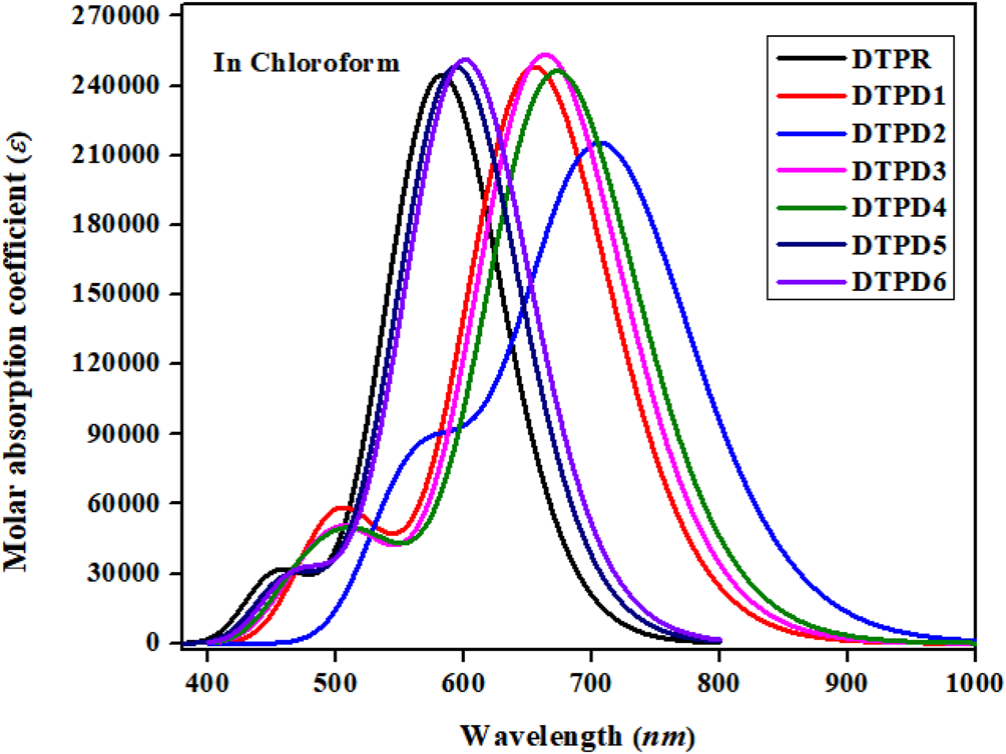
Graphical illustration of UV–Vis absorption spectrum for investigated molecules (DTPR and DTPD1-DTPD6). The UV–Vis graphs are illustrated utilizing Origin Pro 8.5 version (https://www.originlab.com/).

A quantum absorption spectrum is utilized to predict the photoelectric characteristics of compounds in order to ascertain the effectiveness of OSCs^42,43^. Chromophores absorb photons of band gap equivalent energy usually in ultraviolet and visible range of spectrum to get excited. To evaluate the photophysical characteristics of both DTPR and DTPD1-DTPD6, UV/Vis absorption spectra were computed in chloroform solvent. The outcomes of spectral analysis, encompassing parameters such as maximum absorption wavelengths (*λ*_*max*_), oscillator strengths (*f*_os_), excitation energy (*E*_x_ *eV*), as well as transitions nature, are presented in Table 2 (Tables S10) and Fig. 6 showcases the absorption spectra.

**Table 2:** Absorption wavelengths (${\lambda }_{max}$), excitation energy (*E*_x_) and oscillator strength (${f}_{os}$) of DTPR and DTPD1-DTPD6.

Compounds	$\lambda$(*nm*)DFT	$\lambda$(*nm*) Exp	*E*_x_ (*eV*)	*f*_os_	MO contributions
DTPR	585.490	532	2.118	3.182	H-1 → L + 1(11%), H → L(80%)
DTPD1	656.900	-	1.887	3.284	H-1 → L + 1(12%), H → L(79%)
DTPD2	709.693	-	1.747	2.812	H-1 → L + 1(11%), H → L(80%)
DTPD3	665.397	-	1.863	3.350	H-1 → L + 1(12%), H → L(80%)
DTPD4	675.217	-	1.836	3.229	H-1 → L + 1(12%), H → L(81%)
DTPD5	598.462	-	2.072	3.153	H → L(74%), H → L + 1 (5%)
DTPD6	606.899	-	2.043	3.064	H → L(73%), H → L + 1 (6%)

The outcomes presented in Table 2 show that ${\lambda }_{max}$ of DTPR is determined to be 585.490 nm, demonstrating good concurrence with the experimental value of 532 nm for this molecule. The electron accepting units influenced the ${\lambda }_{\mathrm{max}}$ values affecting the red shift in the absorption spectra^44^. Calculations also revealed that DTPR and all the designed compounds (DTPD1-DTPD6) exhibited absorbance in the visible range of 585–709.693 nm. The ${\lambda }_{\mathrm{max}}$ values of DTPR and DTPD1-DTPD6 are calculated at 585.490, 656.900, 709.693, 665.397, 675.217, 598.462 and 606.899 nm, correspondingly. Minimum ${\lambda }_{\mathrm{max}}$ is exhibited by reference molecule (DTPR) among all the investigated chromophores. The strong electron accepting dicyano group in DTPD1 effectively increase ${\lambda }_{\mathrm{max}}$ at 656.900 nm. The ${\lambda }_{\mathrm{max}}$ value of DTPD2 is spotted highest (709.693 nm). This is due to cyano and acetate groups at the terminal A indicating effectiveness of DTPD2 as compared to all investigated molecules. The ${\lambda }_{\mathrm{max}}$ values of DTPD3 and DTPD4 (665.397 and 675.217 nm) are spotted slightly higher than ${\lambda }_{\mathrm{max}}$ of DTPD1 because of MTM and CTM A moieties in DTPD3 and DTPD4, respectively. Similarly, the ${\lambda }_{\mathrm{max}}$ values for DTPD5 and DTPD6 are found to be 598.462 and 606.899 nm with 3.153 and 3.064 oscillator strength and 74 and 73% HOMO to LUMO contribution, respectively. The ${\lambda }_{\mathrm{max}}$ values of investigated compounds in increasing order are: DTPR < DTPD5 < DTPD6 < DTPD1 < DTPD3 < DTPD4 < DTPD2. Consequently, raise in the strength of electron-attracting moieties causes a consistent enhancement in the maximum absorption value *λ*_max_.

Excitation energy is another significant entity that influence the mobility of electrons. Molecules with lower *E*_x_ values tend to have higher charge transfer. This, in turn, leads to an increased power conversion efficiency (PCE), which ultimately results in better optoelectronic properties. Therefore, it is important to investigate the excitation energy as a fundamental parameter in the analysis of OSCs^45^. DTPR has the highest *E*_x_ value (2.118 eV). The strong electron withdrawing units minimize the excitation energy values in compounds DTPD1-DTPD6. Thus, measured transition energy outcomes manifest that DTPR possess larger value of transition energy than the DTPD1-DTPD6. The *E*_x_ outcome of DTPR and DTPD1-DTPD6 are noticed as 2.11, 1.887, 1.747, 1.863, 1.836, 2.072 and 2.043 eV, correspondingly. The minimum *E*_x_ (1.747 eV) is perceived for studied compound DTPD2 because of cyano and acetate in the acceptor portion. The decreasing order of *E*_x_ for investigated moieties is DTPR > DTPD5 > DTPD6 > DTPD1 > DTPD3 > DTPD4 > DTPD2. This is corresponds to the escalating order of *λ*_max_. DTPD2 has the maximum* λ*_max_ and minimum *E*_x_ among the investigated chromophores and thus have outstanding potential for use in fullerene-free OSCs.

### Reorganization ENERGY (RE)

RE is known as molecular charge transfer capability, is additional imperative feature to disclose the effectiveness of OSCs as well as to evaluate the electronic applications of a material^46^. The potential of PV cell is largely reliant on RE which is fundamentally related to the molecular capacity to transport holes and electrons. Usually, reverse correlation exists between charge mobilities and RE. RE is identified via variety of variables, primarily the geometries of cation and anion. Anionic geometry indicates electron mobility (${\lambda }_{\mathrm{e}.}$) typically out of the D while, cationic geometry expresses hole mobility (${\lambda }_{\mathrm{h}}$) often from the acceptor end. So, the calculation of the transmission of charge from donor to accepter can be done by RE. Moreover, the reorganization is categorized in two parts: internal reorganization energy (${\lambda }_{\mathrm{int}.}$) and external reorganization energy (${\lambda }_{\mathrm{ext}.}$). Internal reorganization is associated with changes in internal geometry. External reorganization is concerned with the polarization effects on the external environment^47^. Here we only deal with the internal RE and neglect the external environmental changes. So, by decreasing the RE of donor moieties, the charge transfer rate is increased^45^. Thus, the evaluation of the charge transfer properties of reference (DTPR) and examined compounds (DTPD1-DTPD6). RE is computed and is presented in Table 3, to calculate reorganization energies for both electron (*λ*_e_) and hole (*λ*_h_), Eqs. (1) and (2) ^48–51^ are utilized.

1 $$\lambda_{e} = \left[ {E_{o}^{ - } - E_{ - } } \right] + \left[ {E_{ - }^{o} - E_{o} } \right]$$

2 $$\lambda_{h} = \left[ {E_{o}^{ + } - E_{ + } } \right] + \left[ {E_{ + }^{o} - E_{o} } \right]$$

**Table 3:** Computed RE of entitled compounds DTPR and DTPD1-DTPD6 with its analogues.

Compounds	${\lambda }_{\mathrm{e}}$	${\lambda }_{\mathrm{h}}$
DTPR	0.00700	0.00889
DTPD1	0.00370	0.00782
DTPD2	0.00326	0.00841
DTPD3	0.00368	0.00780
DTPD4	0.00349	0.00805
DTPD5	0.00645	0.00739
DTPD6	0.00558	0.00786

${\lambda }_{\mathrm{e}}$: Reorganization energy of electron, ${\lambda }_{\mathrm{h}}$: Reorganization energy of hole.

The $\lambda_{{\text{e}}}$ values for DTPDR and DTPD1-DTPD6 are 0.00700, 0.00370, 0.00326, 0.00368, 0.00349, 0.00645 and 0.00558, correspondingly. All the investigated chromophores exhibit lower $\lambda_{{\text{e}}}$ as compared to the reference compound (DTPR). This means that all the studied chromophores have greater electron transport ability than DPTR. The $\lambda_{{\text{e}}}$ value for DTPD2 (0.00326) is lowest among all the studied compounds which indicates that the kinetics of electron transition of DTPD2 is highest among all other investigated compounds. The decreasing trend of $\lambda_{{\text{e}}}$ of investigated moieties is: DTPR > DTPD5 > DTPD6 > DTPD1 > DTPD3 > DTPD4 > DTPD2. The $\lambda_{{\text{h}}}$ outcomes for DTPD1-DTPD6 are 0.00782, 0.00841, 0.00780, 0.00805, 0.00739 and 0.00786, correspondingly, lesser than DTPR (0.00889). This shows that derivative molecules have greater hole transfer ability as compare to reference compound. The descending sequence of $\lambda_{{\text{h}}}$ is: DTPR > DTPD2 > DTPD4 > DTPD6 > DTPD1 > DTPD3 > DTPD5. The $\lambda_{{\text{e}}}$ value for DTPD5 (0.00739) is lowest among all the studied compounds. This indicates rate of electron transmission in DTPD5 is highest among all other investigated compounds. So, from above analysis it is evident that DTPD2 and DTPD5 are suitable molecules for hole and electron transport, respectively, thus suitable for advanced applications for proficient OSCs.

### Open circuit voltage (*V*_oc_)

Open circuit voltage is another substantial method utilized to depict the performance of OSCs^52^. It is the total current offered by an instrument at zero applied voltage^53^. It depends on the HOMO (D) − LUMO (A), carrier creation rate, light intensity, charge mobilities and device temperature. *V*_oc_ mainly relies on saturation current besides generated light that facilitates recombination in instruments. It is stated that addition of D and A in molecules leads to improved *λ*_max_ that induces higher *V*_oc_ and enhanced photon catching capacity. To attain greater *V*_oc_, HOMO of D ought to be lesser whereas, A LUMO level should exhibit greater value^54^. In *V*_oc_ analysis, the LUMO of A is scaled with the HOMO of D. The difference between the two energy orbitals minus an empirical factor (0.3 V) is regarded as the total *V*_oc._ The computational results of the exploration of *V*_oc_ of SCs are computed by following Eq. (3), testified by Scharber and his co-workers^55^.

3 $$V_{{{\text{oc}}}} = \left( {\left| {{\text{E}}_{{{\text{HOMO}}}}^{{\text{D}}} } \right| - \left| {{\text{E}}_{{{\text{LUMO}}}}^{{\text{A}}} } \right|} \right) - 0.3$$

**Figure 7:**
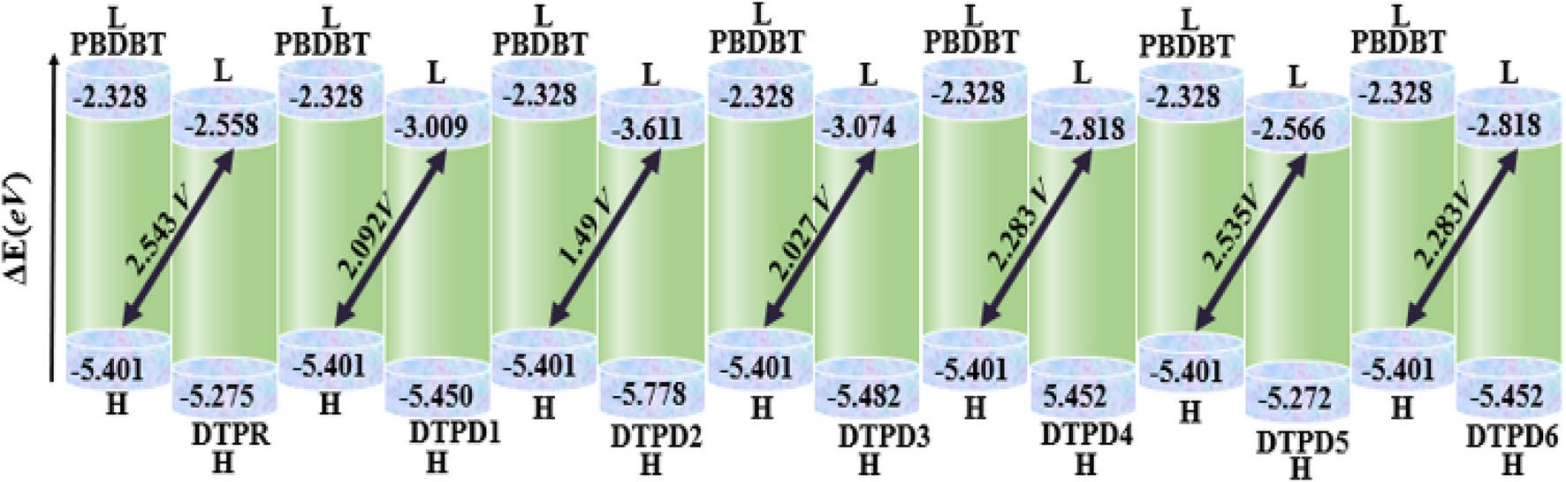
The *V*_oc_ values of DTPR and DTPD1-DTPD6 with reference to PBDBT.

In our study, *V*_oc_ values for DTPR and DTPD1-DTPD6 are calculated by utilizing well-known electron donor polymer PBDBT. The *E*_LUMO_ of DTPR and DTPD1-DTPD6 are observed to be in comparison with the *E*_HOMO_ of PBDBT as displayed in Fig. 7 and the computed values for *V*_oc_ are shown in Table S9. DTPR has *V*_oc_ value of 1.275 V. The designed molecules (DTPD1-DTPD6) disclose *V*_oc_ results as: 2.092, 1.490, 2.027, 2.283, 2.535 and 2.283* V*, respectively. Highest *V*_oc_ is computed in DTPD5, while DTPD2 showed the lowest *V*_oc_ value. Reason behind this is the highest values of HOMO of DTPD2, as mentioned in FMOs analysis discussion. All the derivatives have comparable *V*_oc_ value with the reference molecule (2.543* V*) except DTPD2 (1.490* V*). The ascending trend of *V*_oc_ is: DTPD2 < DTPD3 < DTPD1 < DTPD4 = DTPD6 < DTPD5 < DTPR1. This corresponds to the increasing pattern of HOMO values as given: DTPD5 < DTPR < DTPD1 < DTPD4 = DTPD6 < DTPD3 < DTPD2. The computed outcomes clearly demonstrate that DTPD5 exhibit remarkable properties and can be deemed as probable electron donating materials for OSCs to boost PCE.

### Transition Density Matrix (TDM) and Exciton Binding Energy (*E*_b_)

**Figure 8:**
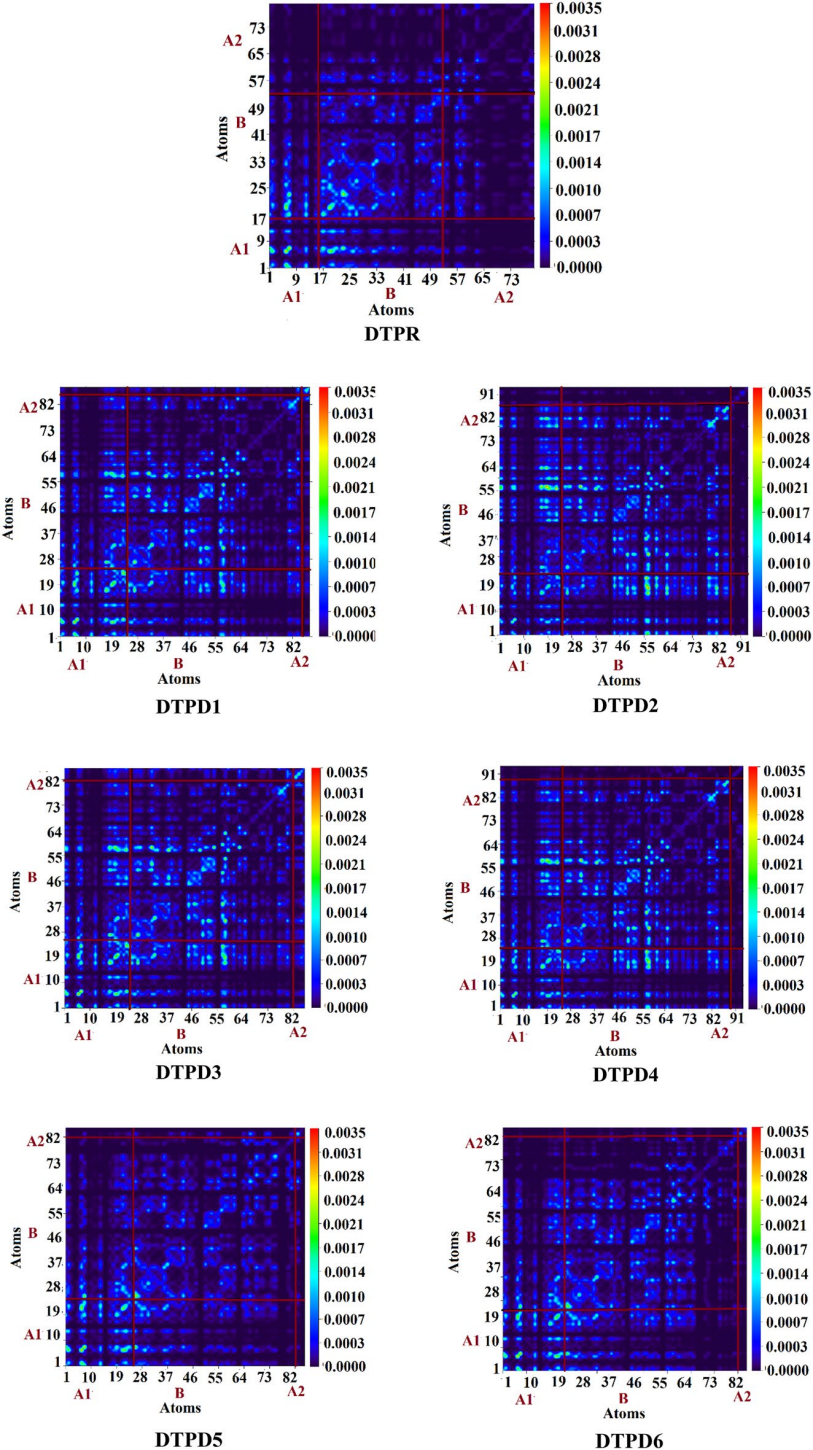
Pictorial illustration of TDM plots of DTPR and DTPD1-DTPD6. The TDM pictographs are drawn utilizing Multiwfn version.

TDM analysis is performed to estimate the transitions type in all the computed compounds (DTPR and DTPD1-DTPD6). Types of connections among D and A are determined through TDM. It also determine the electron–hole location within the compound as well as electron excitation state^59,60^. In our work, hydrogen atoms effect in all the investigated chromophores is neglected which is because of the small contribution of hydrogen atom in electronic transmission. To inspect variant interactions, DTPR and DTPD1-DTPD6) molecules are distributed into three parts; i) acceptor 1 labeled as A1, ii) *π*-spacer labeled as B and iii) acceptor 2 group as A2. TDM plots illustrate that the DTPR, DTPD5 and DTPD6 similar response where electron density is located over acceptor 1. Minimal charge is also present over the *π*-spacer region. Similarly, DTPD1 and DTPD4 exhibit almost same behavior. The electron density of the foresaid moieties is located on end-capped acceptor groups (A1 and A2) and minutely on *π*-spacer region. The electron density of the DTPD2 and DTPD3 is completely contained by A1 and A2. Almost no charge coherence is present on *π*-spacer region. This is supported by the fact that the mentioned investigated chromophores (DTPD2 and DTPD3) have least energy band gap as discussed in FMOs analysis. This means that the foresaid chromophores has maximum bathochromic shift. As discussed in “Optical Properties” section DTPD2 showed the maximum *λ*_max_ peak, exhibiting the maximum charge transfer towards acceptor moieties. The heat maps verify efficient migration of electrons from the *π*-spacer region to A1 and A2 in all the molecules as shown in Fig. 8. DOS is also reinforced by these heat maps, which equally manifests the charge transmission.

The binding energy (*E*_b_) is substantial to decide the molecular optoelectronic characteristics. Chromophores absorb photons and go to exciton state^61^. Conjugated molecules manifest strong attractive coulombic forces among the charge carriers because of less dielectric constant. The coulombic force of interaction present among hole—electron is calculated via binding energy^62^. Introduction of “A” at the terminals help in reducing coulombic forces and subsequently easier dissociation^63^. Coulombic attractions have direct relation with binding energy, but a reversed relation is detected among *E*_b_ and exciton dissociation. The *E*_b_ value of DTPR and DTPD1-DTPD6 molecules are calculated by using the following Eq. ^64^.

4 $$E_{{\text{b}}} = E_{{{\text{L}} - {\text{H}}}} - E_{{{\text{opt}}}}$$

where $E_{{{\text{L}} - {\text{H}}}}$ represents energy band gap between HOMO to LUMO. Optical energy ($E_{{{\text{opt}}}}$) is the minimum amount of energy required for the first excitation which is secured from S_0_ to S_1_, first singlet excited state energy by pairing of the electron and hole^65^. Moreover, the charge dissociation capability of DTPD2, DTPD3, DTPD4 and DTPD6 molecules are also found higher than DTPR, DTPD1 and DTPD5 which suggests that the earlier mentioned molecules (DTPD2, DTPD3, DTPD4 and DTPD6) would profoundly boost the whole current charge density as compared to the DTPR, DTPD1 and DTPD5. DFT based computed outcomes gained for *E*_b_ of all investigated molecules are tabulated in Table 4.

**Table 4:** Calculated ${E}_{L-H}$, ${E}_{opt}$ and ${E}_{b}$ of investigated chromophores (DTPR and DTPD1-DTPD6).

Molecules	${E}_{\mathrm{L}-\mathrm{H}}$(*eV*)	${E}_{\mathrm{opt}}$(*eV*)	${E}_{\mathrm{b}}$(*eV*)
DTPR	2.717	2.118	0.599
DTPD1	2.441	1.887	0.554
DTPD2	2.167	1.747	0.420
DTPD3	2.408	1.863	0.545
DTPD4	2.634	1.836	0.798
DTPD5	2.706	2.072	0.634
DTPD6	2.634	2.043	0.591

Table 4 demonstrates that the *E*_b_ values for aforesaid molecules (DTPR andDTPD1-DTPD6) are 0.599, 0.554, 0.420, 0.545, 0.798, 0.634 and 0.591 eV, respectively. DTPD2 showed lowest value of *E*_b_ among all the examined molecules which demonstrated its greater exciton dissociation rate in comparison to the other chromophores. The decreasing trend of *E*_b_ values of all the examined molecules is found as: DTPD6 > DTPD4 > DTPD2 > DTPD3 > DTPD1 = DTPD5 > DTPR. These compounds have recognized to be excellent candidates for fullerene free OSCs as they yield maximum amount of voltage.

## Conclusion

In summary, BDD-IN based organic chromophores with A_2_-*π*-A_1_-*π*-A_2_ architecture has been quantum chemically designed by utilizing end-capped modulation approach to make competent photovoltaic materials. Different kinds of robust electron-withdrawing groups have been introduced by structural modification of terminal acceptors to acquire larger red-shift with reduced energy gap. A narrow down energy gap (2.167–2.717 eV) along with wider absorption spectra (585.490–709.693 nm) was observed in DTPD1-DTPD6 as compared to the DTPR chromophore. Moreover, the *V*_oc_ results depicted that with respect to HOMO_PBDBT_ –LUMO_acceptor_, all the compounds illustrated comparable voltage. Interestingly, DTPD2 demonstrated the lowest band gap (2.167 eV) with wider absorption spectra (709.693) and lowest transition energy values (1.747 eV) among all the titled compounds which is attributed to the strong electron accepting terminal moiety. Thus, bridging core modulation is demonstrated to be an efficient strategy with improved optical as well as electronic characteristics for efficient OSCs.

### Supplementary Information

Supplementary Information.

## Data Availability

All data generated and analyzed during this study are included in this published article and its supplementary information files.
