# Supplementary material for: Designing of banana shaped chromophores via molecular engineering of terminal groups to probe photovoltaic behavior of organic solar cell materials
Source: Sci Rep. 2023 Sep 12;13:15064. doi: 10.1038/s41598-023-39496-6 (PMC10497593; doi:10.1038/s41598-023-39496-6)
Supplement: Supplementary file 1 — Supplementary Information. [file 41598_2023_39496_MOESM1_ESM.docx]

**Designing of Banana Shaped Chromophores *via* Molecular Engineering of Terminal Groups to Probe Photovoltaic Behavior of Organic Solar Cell Materials**

Saeed Ahmed^1,2^, Iram Irshad^2,3^ Saima Nazir,^4,5^ Salma Naz, ^2,3^ Muhammad Adnan Asghar^6^, Saad M. Alshehri,^7^ Saifullah Bullo,^8^ Muhammed Lamin Sanyang,*^9^

^1^Department of Pharmaceutical Sciences, University of Milan, Via Venezian 21, 20133 Milan, Italy

^2^Institute of Chemistry, Khwaja Fareed University of Engineering & Information Technology, Rahim Yar Khan, 64200, Pakistan

^3^Centre for Theoretical and Computational Research, Khwaja Fareed University of Engineering & Information Technology, Rahim Yar Khan, 64200, Pakistan

^4^ Nawaz Sharif Medical College, University of Gujrat, Pakistan

^5^Institute of Biological Sciences, Khwaja Fareed University of Engineering & Information Technology, Rahim Yar Khan, 64200, Pakistan

^6^Department of Chemistry, Division of Science and Technology, University of Education Lahore, Pakistan

^7^Department of Chemistry, College of Science, King Saud University, Saudi Arabia,

^8^Department of Human and Rehabilitation Sciences, Begum Nusrat Bhutto Women University, Sukkur Sindh Pakistan

^9^Directorate of Research and Consultancy, University of The Gambia, Kanifing Campus, MDI Road, P.O Box 3530, The Gambia

*Corresponding author E-mail addresses:

Dr. Muhammed Lamin Sanyang (m.sanyang@utg.edu.gm)

**Table S1:** Cartesian coordinates of **DTPR**

| **Atoms** | **X-axis** | **Y-axis** | **Z-axis** |
| --- | --- | --- | --- |
| C | -0.45723 | 0.152778 | 0.570042 |
| C | 1.096318 | 0.136638 | 0.622076 |
| C | 1.871008 | 1.00006 | 1.639351 |
| C | 1.034118 | 1.902504 | 2.573564 |
| C | -0.51108 | 1.923374 | 2.50395 |
| C | -1.2863 | 1.043256 | 1.508271 |
| C | -1.06323 | 2.771514 | 3.405335 |
| C | 1.520545 | 2.73694 | 3.529288 |
| C | 1.633702 | -0.71249 | -0.30025 |
| C | -0.95443 | -0.70112 | -0.36177 |
| O | 3.127455 | 0.970984 | 1.703711 |
| O | -2.5447 | 1.05168 | 1.46197 |
| S | 0.205345 | 3.718404 | 4.210454 |
| S | 0.361242 | -1.32261 | -1.37031 |
| C | 2.99684 | 2.816748 | 3.959106 |
| H | 3.456502 | 1.858408 | 3.834098 |
| H | 3.504974 | 3.53771 | 3.353494 |
| H | 3.05593 | 3.108731 | 4.986664 |
| C | -2.5754 | 2.885556 | 3.68438 |
| H | -3.00094 | 3.62026 | 3.03369 |
| H | -3.04242 | 1.939015 | 3.509114 |
| H | -2.73299 | 3.176904 | 4.702176 |
| C | -2.43384 | -1.10361 | -0.53657 |
| C | -2.85293 | -2.0389 | -1.42942 |
| S | -3.81282 | -0.4448 | 0.365916 |
| C | -4.39529 | -2.13054 | -1.50792 |
| H | -2.19109 | -2.63907 | -2.01788 |
| C | -5.00553 | -1.24079 | -0.68178 |
| H | -4.9196 | -2.80743 | -2.14858 |
| C | 3.113387 | -1.12815 | -0.44076 |
| C | 3.562922 | -1.90524 | -1.45906 |
| S | 4.454653 | -0.69665 | 0.641283 |
| C | 5.105223 | -2.035 | -1.47378 |
| H | 2.924121 | -2.36754 | -2.17823 |
| C | 5.689199 | -1.35988 | -0.45258 |
| H | 5.650511 | -2.58817 | -2.21052 |
| C | -6.52332 | -0.97854 | -0.68487 |
| C | -7.0862 | -0.00804 | 0.157843 |
| C | -7.3422 | -1.72174 | -1.54584 |
| C | -8.4722 | 0.227876 | 0.119114 |
| C | -8.7212 | -1.49914 | -1.57307 |
| H | -6.9109 | -2.46395 | -2.18454 |
| C | -9.28792 | -0.52365 | -0.7459 |
| H | -8.90555 | 0.978473 | 0.748156 |
| C | 7.216738 | -1.21165 | -0.28667 |
| C | 8.069293 | -1.67641 | -1.29772 |
| C | 7.756238 | -0.62422 | 0.867075 |
| C | 9.458673 | -1.5888 | -1.14165 |
| H | 7.657238 | -2.09991 | -2.18978 |
| C | 9.149395 | -0.55993 | 1.035362 |
| C | 9.999338 | -1.06124 | 0.037388 |
| H | 9.561987 | -0.13093 | 1.923622 |
| O | -6.24602 | 0.734651 | 1.048098 |
| O | 6.887934 | -0.10033 | 1.87584 |
| O | 10.3282 | -2.04191 | -2.18219 |
| O | -9.55711 | -2.26381 | -2.44638 |
| C | 9.692463 | -3.08537 | -2.92431 |
| H | 9.469768 | -3.903 | -2.27184 |
| H | 8.785663 | -2.71546 | -3.35493 |
| H | 10.34681 | -3.41802 | -3.70281 |
| C | -6.98029 | 1.119235 | 2.212828 |
| H | -6.34017 | 1.659996 | 2.8791 |
| H | -7.80242 | 1.740517 | 1.926802 |
| H | -7.34845 | 0.244678 | 2.706062 |
| C | 7.51368 | -0.22081 | 3.156042 |
| H | 8.433716 | 0.325392 | 3.156647 |
| H | 6.863429 | 0.174077 | 3.90802 |
| H | 7.711657 | -1.25261 | 3.36137 |
| C | 11.52932 | -1.04306 | 0.227224 |
| C | 12.42228 | -1.43759 | -0.71816 |
| S | 12.37184 | -0.52617 | 1.699941 |
| C | 13.8687 | -1.51095 | -0.16391 |
| H | 12.15683 | -1.68124 | -1.72501 |
| C | 13.93106 | -1.17196 | 1.150251 |
| H | 14.71707 | -1.80761 | -0.74526 |
| C | -10.8109 | -0.29329 | -0.7984 |
| C | -11.4901 | 0.640766 | -0.08237 |
| S | -11.9213 | -1.22579 | -1.81665 |
| C | -12.987 | 0.710748 | -0.48616 |
| H | -11.0449 | 1.263066 | 0.663964 |
| C | -13.2996 | -0.17359 | -1.47015 |
| H | -13.6915 | 1.383894 | -0.0496 |
| C | 15.18911 | -1.34304 | 2.0252 |
| C | 15.16788 | -1.02945 | 3.343334 |
| H | 16.091 | -1.71882 | 1.585953 |
| C | 13.98512 | -0.4852 | 4.155482 |
| C | 16.33727 | -1.15616 | 4.328493 |
| C | 14.45543 | 0.224843 | 7.946758 |
| C | 15.81679 | -0.1645 | 8.043742 |
| C | 16.50984 | -0.62783 | 6.906903 |
| C | 15.81732 | -0.68989 | 5.701747 |
| C | 14.48379 | -0.30873 | 5.6051 |
| C | 13.77714 | 0.153293 | 6.711452 |
| H | 13.94006 | 0.574755 | 8.816929 |
| H | 16.32095 | -0.10619 | 8.985117 |
| H | 17.53619 | -0.92342 | 6.968844 |
| H | 12.75138 | 0.444958 | 6.628493 |
| O | 12.83043 | -0.2318 | 3.722764 |
| O | 17.49811 | -1.56345 | 4.06505 |
| C | -14.6626 | -0.25536 | -2.18554 |
| C | -14.8628 | -1.174 | -3.15953 |
| H | -15.4534 | 0.410324 | -1.91209 |
| C | -13.8284 | -2.15542 | -3.72448 |
| C | -16.1672 | -1.44728 | -3.91981 |
| C | -16.2481 | -4.23054 | -6.62258 |
| C | -14.8886 | -4.62687 | -6.52584 |
| C | -14.025 | -3.99669 | -5.60835 |
| C | -14.5479 | -2.9855 | -4.80613 |
| C | -15.8737 | -2.59065 | -4.90895 |
| C | -16.7487 | -3.19561 | -5.80372 |
| H | -16.8957 | -4.71818 | -7.31886 |
| H | -14.5167 | -5.40875 | -7.15247 |
| H | -12.9994 | -4.28569 | -5.53009 |
| H | -17.7695 | -2.88659 | -5.86656 |
| O | -17.2672 | -0.85436 | -3.765 |
| O | -12.6236 | -2.26235 | -3.37477 |
| C | -8.8282 | -2.62249 | -3.62379 |
| H | -8.50718 | -1.73522 | -4.13041 |
| H | -7.97298 | -3.20657 | -3.35198 |
| H | -9.45907 | -3.19456 | -4.27216 |

**Table S2:** Cartesian coordinates of **DTPD1**

| **Atoms** | **X-axis** | **Y-axis** | **Z-axis** |
| --- | --- | --- | --- |
| C | -0.70385000 | 2.66170000 | -0.28459300 |
| C | 0.73942600 | 2.64795300 | -0.24497200 |
| C | 1.53044100 | 3.86936900 | -0.45904900 |
| C | 0.77232800 | 5.06057600 | -0.85427000 |
| C | -1.45835100 | 3.89887500 | -0.53376800 |
| C | -1.28057300 | 1.41141700 | -0.10716200 |
| C | 1.28149100 | 1.38785300 | -0.03327600 |
| C | -0.65815200 | 5.07486000 | -0.88795000 |
| C | -1.18707800 | 6.28496400 | -1.26281800 |
| C | 1.34134700 | 6.26003300 | -1.20432300 |
| S | 0.09647600 | 7.40760800 | -1.58739400 |
| S | -0.01824900 | 0.23602700 | 0.18131400 |
| C | -2.61027700 | 6.69548100 | -1.39953400 |
| H | -3.17318700 | 5.94161900 | -1.95807800 |
| H | -3.08729700 | 6.76576200 | -0.41505500 |
| H | -2.70357600 | 7.66387500 | -1.90070800 |
| C | 2.77642800 | 6.64451200 | -1.27655500 |
| H | 3.21352100 | 6.69725100 | -0.27289000 |
| H | 3.34924500 | 5.88696600 | -1.81961700 |
| H | 2.90819600 | 7.61557900 | -1.76379000 |
| O | -2.68215200 | 3.96023200 | -0.45136900 |
| O | 2.74966900 | 3.90519200 | -0.31712100 |
| C | -2.62448400 | 0.87576600 | -0.13092200 |
| C | -4.26435200 | -0.78552100 | -0.18917100 |
| C | -5.07128300 | 0.32174200 | 0.01049900 |
| H | -4.64183600 | -1.79622300 | -0.30786400 |
| C | 2.61489400 | 0.82797500 | 0.02263800 |
| C | 2.87241700 | -0.53291300 | -0.05866800 |
| H | 2.09813600 | -1.27160400 | -0.24709400 |
| C | 4.21778100 | -0.86873100 | 0.10936600 |
| C | 5.03416900 | 0.22663400 | 0.32791200 |
| H | 4.57744200 | -1.89247700 | 0.07796600 |
| S | 4.10142000 | 1.69937300 | 0.31493600 |
| S | -4.10825800 | 1.77524600 | 0.06792600 |
| C | -2.90457000 | -0.47653200 | -0.26481400 |
| H | -2.13686500 | -1.22488700 | -0.44196800 |
| C | -6.52465300 | 0.30785300 | 0.12981400 |
| C | -7.31207500 | 1.48047200 | 0.16969500 |
| C | -7.20802600 | -0.91403500 | 0.19838400 |
| C | -8.69143300 | 1.40439600 | 0.26260400 |
| C | -8.59027300 | -0.99149700 | 0.28653400 |
| H | -6.63174300 | -1.83222500 | 0.19167200 |
| C | -9.37560500 | 0.18048100 | 0.32714300 |
| H | -9.26776100 | 2.32262000 | 0.26123400 |
| C | 6.48563300 | 0.18945100 | 0.46304500 |
| C | 7.25068600 | 1.28179500 | 0.92932500 |
| C | 7.18414800 | -0.97438100 | 0.11767400 |
| C | 8.62618300 | 1.17744500 | 1.04968600 |
| C | 8.56265900 | -1.07930200 | 0.23691000 |
| H | 6.62508300 | -1.81246100 | -0.28356100 |
| C | 9.32542700 | 0.00572400 | 0.71951200 |
| H | 9.19023100 | 2.03855000 | 1.38960300 |
| O | -6.63839200 | 2.65261300 | 0.10094900 |
| O | 6.56014900 | 2.40690800 | 1.23149100 |
| O | 9.25370200 | -2.19138600 | -0.09941200 |
| O | -9.26375900 | -2.16245800 | 0.34526400 |
| C | 7.26048300 | 3.54491900 | 1.67103000 |
| H | 7.79067500 | 3.35219200 | 2.61418900 |
| H | 7.98002500 | 3.89277000 | 0.91683000 |
| H | 6.51010500 | 4.32006600 | 1.83373500 |
| C | -7.35439000 | 3.86324400 | 0.11198600 |
| H | -8.04582200 | 3.92953600 | -0.73942900 |
| H | -7.91843200 | 3.98902100 | 1.04659700 |
| H | -6.60928600 | 4.65658600 | 0.03168800 |
| C | 10.77134900 | -0.03721500 | 0.87627100 |
| C | 11.53471700 | 0.92842500 | 1.52863900 |
| S | 11.77979300 | -1.30113100 | 0.22831500 |
| C | 12.89624400 | 0.66438300 | 1.49272900 |
| H | 11.11023500 | 1.79067500 | 2.03136800 |
| C | 13.22992400 | -0.51024500 | 0.81545800 |
| H | 13.65693200 | 1.29491300 | 1.94659500 |
| C | -10.82646300 | 0.17012800 | 0.43399700 |
| C | -11.61939200 | 1.28659000 | 0.69056300 |
| S | -11.80329700 | -1.25402200 | 0.20569100 |
| C | -12.97765500 | 1.00389300 | 0.68558700 |
| H | -11.21911200 | 2.27428500 | 0.89278700 |
| C | -13.27961300 | -0.33478900 | 0.42739300 |
| H | -13.75876600 | 1.73806000 | 0.86771200 |
| C | -8.54850000 | -3.37379300 | 0.28454200 |
| H | -9.29360600 | -4.16899700 | 0.33402600 |
| H | -7.98661500 | -3.46046300 | -0.65543100 |
| H | -7.85529300 | -3.47302200 | 1.13093900 |
| C | 8.56195400 | -3.31215900 | -0.59733400 |
| H | 7.83510700 | -3.69161900 | 0.13395500 |
| H | 8.04254500 | -3.07968400 | -1.53694800 |
| H | 9.31786800 | -4.07631700 | -0.78383100 |
| C | 14.58804200 | -0.89354900 | 0.70355300 |
| H | 15.22317300 | -0.15942800 | 1.19547100 |
| C | -14.63127800 | -0.75416800 | 0.39207300 |
| H | -15.28818600 | 0.09182800 | 0.58445200 |
| C | 15.23894400 | -1.95316800 | 0.12174400 |
| C | 14.59325600 | -3.06956600 | -0.59392800 |
| C | 16.68040400 | -2.18133700 | 0.09863800 |
| C | 15.67676300 | -3.96628800 | -1.03684300 |
| C | -15.25369500 | -1.95675300 | 0.16439200 |
| C | -14.57265500 | -3.23162500 | -0.12645200 |
| C | -16.69220800 | -2.20523800 | 0.14951500 |
| C | -15.63156300 | -4.23884600 | -0.32329500 |
| C | 16.91278600 | -3.44957900 | -0.62768000 |
| C | 18.07680800 | -4.15149000 | -0.94007300 |
| C | 15.55676500 | -5.14866800 | -1.74284100 |
| C | 17.96093100 | -5.34432600 | -1.65092800 |
| H | 19.05964900 | -3.79954500 | -0.64682000 |
| C | 16.72150200 | -5.84345800 | -2.05212000 |
| H | 14.57157500 | -5.50326000 | -2.03615300 |
| H | 18.86384200 | -5.89710300 | -1.89787400 |
| H | 16.67138700 | -6.77711200 | -2.60660800 |
| C | -16.88685500 | -3.63894500 | -0.16158100 |
| C | -18.03406400 | -4.41872900 | -0.30753900 |
| C | -15.47563300 | -5.57908100 | -0.62352300 |
| C | -17.88214000 | -5.77020800 | -0.61070400 |
| H | -19.03048500 | -4.00700200 | -0.19295100 |
| C | -16.62375000 | -6.35122300 | -0.76855100 |
| H | -14.47690000 | -5.99329800 | -0.73914600 |
| H | -18.77168200 | -6.38424000 | -0.72613600 |
| H | -16.54562600 | -7.40926100 | -1.00539600 |
| O | 13.40178600 | -3.24251700 | -0.79203300 |
| O | -13.37248700 | -3.43971500 | -0.19737700 |
| C | 17.44792900 | -0.17224700 | 1.32588500 |
| C | 19.06345700 | -1.72626700 | 0.53635500 |
| C | 17.67939100 | -1.39573300 | 0.63053900 |
| N | 17.28201600 | 0.82940600 | 1.89701200 |
| N | 20.20146700 | -1.96570100 | 0.47677000 |
| C | -17.71672400 | -1.31227800 | 0.37537500 |
| C | -17.51957000 | 0.06714700 | 0.67866800 |
| C | -19.09418500 | -1.67827600 | 0.32856700 |
| N | -20.22775400 | -1.94271400 | 0.29697900 |
| N | -17.38110400 | 1.19674300 | 0.92734900 |

**Table S3:** Cartesian coordinates of **DTPD2**

| **Atom** | **X-axis** | **Y-axis** | **Z-axis** |
| --- | --- | --- | --- |
| C | -0.70309300 | -3.69743000 | 0.35365500 |
| C | 0.73739700 | -3.67380300 | 0.26581000 |
| C | 1.54305300 | -4.89240600 | 0.44119400 |
| C | 0.80505200 | -6.09328400 | 0.84172900 |
| C | -1.44203400 | -4.94121100 | 0.62007700 |
| C | -1.29282300 | -2.44923000 | 0.20760900 |
| C | 1.26374000 | -2.40784900 | 0.05134000 |
| C | -0.62305500 | -6.11697100 | 0.92740300 |
| C | -1.13057800 | -7.33697700 | 1.29985700 |
| C | 1.39362900 | -7.29552700 | 1.14686800 |
| S | 0.17088600 | -8.45751900 | 1.55528200 |
| S | -0.04855600 | -1.26296600 | -0.11058100 |
| C | -2.54522000 | -7.75870400 | 1.48186700 |
| H | -3.08979200 | -7.02022500 | 2.07783500 |
| H | -3.05904600 | -7.81071800 | 0.51495900 |
| H | -2.61460400 | -8.73797400 | 1.96535800 |
| C | 2.83351700 | -7.66850300 | 1.15886600 |
| H | 3.25679700 | -7.60033400 | 0.15079800 |
| H | 3.40773600 | -6.97034800 | 1.77637400 |
| H | 2.98261300 | -8.68477200 | 1.53617600 |
| O | -2.66795000 | -5.00596400 | 0.58745700 |
| O | 2.75796700 | -4.91735100 | 0.26432100 |
| C | -2.63861900 | -1.92197600 | 0.27945800 |
| C | -4.28077500 | -0.26738700 | 0.40356700 |
| C | -5.08885300 | -1.37527600 | 0.21174000 |
| H | -4.65913700 | 0.73936100 | 0.54949500 |
| C | 2.59131600 | -1.83794500 | -0.03825300 |
| C | 2.84223100 | -0.47705100 | 0.06303700 |
| H | 2.07028100 | 0.25141500 | 0.29509500 |
| C | 4.17845600 | -0.12836000 | -0.14579000 |
| C | 4.99248000 | -1.21340600 | -0.41777600 |
| H | 4.53346600 | 0.89673900 | -0.10630200 |
| S | 4.07062400 | -2.69287500 | -0.40289800 |
| S | -4.12355000 | -2.82521300 | 0.11200500 |
| C | -2.91897300 | -0.57232500 | 0.43837900 |
| H | -2.14922400 | 0.17604800 | 0.60566900 |
| C | -6.54439400 | -1.36033200 | 0.13873800 |
| C | -7.33669800 | -2.53167600 | 0.14287100 |
| C | -7.22479400 | -0.13796800 | 0.06742400 |
| C | -8.71772100 | -2.45149300 | 0.11624900 |
| C | -8.60917900 | -0.05621800 | 0.03369800 |
| H | -6.64560800 | 0.77742400 | 0.02621300 |
| C | -9.40201700 | -1.22465600 | 0.07328300 |
| H | -9.29329800 | -3.36979000 | 0.12355600 |
| C | 6.43748000 | -1.16154800 | -0.60126300 |
| C | 7.19085900 | -2.22844100 | -1.14258300 |
| C | 7.14063200 | -0.00829100 | -0.23359500 |
| C | 8.55864800 | -2.10765200 | -1.31343500 |
| C | 8.51295000 | 0.11306900 | -0.40205400 |
| H | 6.59288600 | 0.80648900 | 0.22683600 |
| C | 9.26429600 | -0.94485800 | -0.95962500 |
| H | 9.10965400 | -2.94559200 | -1.72446200 |
| O | -6.66217400 | -3.70434300 | 0.17697000 |
| O | 6.49442800 | -3.34332800 | -1.46608700 |
| O | 9.20681400 | 1.21502800 | -0.04519400 |
| O | -9.27644400 | 1.11539900 | -0.03792400 |
| C | 7.18386300 | -4.45872700 | -1.97622100 |
| H | 7.67601800 | -4.22626900 | -2.93088200 |
| H | 7.93372000 | -4.82930700 | -1.26350700 |
| H | 6.43243600 | -5.23256000 | -2.13972700 |
| C | -7.38259100 | -4.91248900 | 0.21055600 |
| H | -8.02892200 | -4.96915200 | 1.09739800 |
| H | -7.99543400 | -5.04034600 | -0.69242800 |
| H | -6.63828000 | -5.70895700 | 0.25651100 |
| C | 10.70121300 | -0.88294300 | -1.16688500 |
| C | 11.46266000 | -1.85979400 | -1.81167800 |
| S | 11.70443100 | 0.42954100 | -0.61572600 |
| C | 12.81420000 | -1.56231600 | -1.84953700 |
| H | 11.04372800 | -2.75849600 | -2.25039000 |
| C | 13.14817300 | -0.34844700 | -1.23908100 |
| H | 13.57008000 | -2.19550800 | -2.30769100 |
| C | -10.85470400 | -1.20354400 | 0.08071500 |
| C | -11.67829500 | -2.32543000 | 0.20053900 |
| S | -11.79903900 | 0.25349100 | -0.05188200 |
| C | -13.02775600 | -2.01597300 | 0.19141700 |
| H | -11.30546900 | -3.33836200 | 0.30579100 |
| C | -13.29954400 | -0.64940000 | 0.06110000 |
| H | -13.82565700 | -2.74871000 | 0.28464900 |
| C | -8.55645600 | 2.32595100 | -0.08998000 |
| H | -9.30271300 | 3.11849600 | -0.15943800 |
| H | -7.95396100 | 2.47209500 | 0.81645700 |
| H | -7.90338100 | 2.36421300 | -0.97201800 |
| C | 8.52650600 | 2.31540300 | 0.51415400 |
| H | 7.77219600 | 2.71169700 | -0.17887400 |
| H | 8.04382400 | 2.04801800 | 1.46361300 |
| H | 9.28458100 | 3.07801500 | 0.69746900 |
| C | 14.49475500 | 0.06963100 | -1.20510300 |
| H | 15.12812600 | -0.66869800 | -1.69297600 |
| C | -14.63522400 | -0.19510200 | 0.06554800 |
| H | -15.31780700 | -1.03524800 | 0.17603500 |
| C | 15.14507200 | 1.17364400 | -0.69889200 |
| C | 14.50064300 | 2.30037100 | -0.00632800 |
| C | 16.57669200 | 1.43394800 | -0.75471600 |
| C | 15.58111600 | 3.24670700 | 0.34525600 |
| C | -15.22303800 | 1.04795200 | -0.03296300 |
| C | -14.50016700 | 2.31488200 | -0.21937400 |
| C | -16.64884700 | 1.34405900 | 0.01630800 |
| C | -15.52557000 | 3.37654500 | -0.28360500 |
| C | 16.80878800 | 2.74216300 | -0.09588800 |
| C | 17.96152300 | 3.48561700 | 0.12942300 |
| C | 15.46579600 | 4.45542500 | 0.99745400 |
| C | 17.86069600 | 4.72000700 | 0.79162800 |
| H | 18.94453100 | 3.15378900 | -0.18862900 |
| C | 16.61255400 | 5.21251800 | 1.23094600 |
| H | 14.49990800 | 4.82668000 | 1.33083800 |
| C | -16.79672000 | 2.81246800 | -0.13912900 |
| C | -17.90714600 | 3.64885900 | -0.16175900 |
| C | -15.32492400 | 4.72864600 | -0.45622600 |
| C | -17.72016700 | 5.03075200 | -0.33250500 |
| H | -18.92122800 | 3.27938700 | -0.05307500 |
| C | -16.42821200 | 5.57951900 | -0.48389700 |
| H | -14.32463300 | 5.13852700 | -0.57132700 |
| O | 13.31758000 | 2.45713000 | 0.24272000 |
| O | -13.29781900 | 2.49445200 | -0.31154900 |
| C | 17.34600800 | -0.60320600 | -1.92721300 |
| C | 18.95074900 | 1.02833900 | -1.27867300 |
| C | 17.57494100 | 0.65505000 | -1.29618500 |
| N | 17.17848600 | -1.63216900 | -2.44651400 |
| N | 20.08163800 | 1.30509300 | -1.27928100 |
| C | -17.70584000 | 0.47605200 | 0.17613100 |
| C | -17.55716700 | -0.93392000 | 0.32847200 |
| C | -19.06595500 | 0.90285600 | 0.20717500 |
| N | -20.18507900 | 1.22236700 | 0.23718600 |
| N | -17.45327700 | -2.08700200 | 0.45478700 |
| C | -18.92271000 | 5.81297500 | -0.34172100 |
| N | -19.95249600 | 6.35126800 | -0.33399400 |
| C | 19.10052300 | 5.41858200 | 0.97279700 |
| N | 20.15427200 | 5.89955500 | 1.06487300 |
| C | -16.12816300 | 7.02860500 | -0.68151700 |
| O | -15.00774200 | 7.45630300 | -0.83555500 |
| O | -17.22376500 | 7.78402000 | -0.66926200 |
| C | 16.40400200 | 6.51005000 | 1.93967000 |
| O | 15.31467400 | 6.90878500 | 2.28088100 |
| O | 17.54241700 | 7.16647300 | 2.15029200 |
| C | -17.01346800 | 9.18182700 | -0.86364200 |
| H | -16.35828600 | 9.58122200 | -0.08446000 |
| H | -16.55293100 | 9.36435900 | -1.83875000 |
| H | -18.00047100 | 9.64002100 | -0.81086300 |
| C | 17.41670900 | 8.42022000 | 2.81976600 |
| H | 16.78562000 | 9.09903700 | 2.23923500 |
| H | 16.96859800 | 8.28124700 | 3.80777400 |
| H | 18.42910100 | 8.81322400 | 2.90641400 |

**Table S4:** Cartesian coordinates of **DTPD3**

| **Atom** | **X-axis** | **Y-axis** | **Z-axis** |
| --- | --- | --- | --- |
| C | 0.69783800 | 2.68476300 | 0.30563000 |
| C | -0.74494500 | 2.66509400 | 0.24807900 |
| C | -1.54272500 | 3.88615400 | 0.43807000 |
| C | -0.79328600 | 5.08701200 | 0.81749300 |
| C | 1.44512100 | 3.92649800 | 0.55668700 |
| C | 1.28097900 | 1.43487300 | 0.14710500 |
| C | -1.27981600 | 1.40063300 | 0.04384600 |
| C | 0.63588700 | 5.10641200 | 0.87365200 |
| C | 1.15538800 | 6.32794000 | 1.22362800 |
| C | -1.37173700 | 6.29355100 | 1.12514200 |
| S | -0.13665000 | 7.45458300 | 1.49714400 |
| S | 0.02644700 | 0.25178900 | -0.14398200 |
| C | 2.57467600 | 6.74579300 | 1.37649700 |
| H | 3.09919900 | 6.08132500 | 2.07068000 |
| H | 3.10316900 | 6.66680900 | 0.42023500 |
| H | 2.65358100 | 7.77480300 | 1.74032600 |
| C | -2.80963300 | 6.67260400 | 1.16356200 |
| H | -3.25493400 | 6.59484500 | 0.16570600 |
| H | -3.37340400 | 5.98458900 | 1.80167200 |
| H | -2.94630600 | 7.69385500 | 1.53218700 |
| O | 2.67050800 | 3.98912100 | 0.50478400 |
| O | -2.76127000 | 3.91560500 | 0.28857100 |
| C | 2.62693500 | 0.90497000 | 0.18608400 |
| C | 4.27274000 | -0.74961400 | 0.26171600 |
| C | 5.07592400 | 0.35933400 | 0.05610800 |
| H | 4.65410300 | -1.75778900 | 0.38928000 |
| C | -2.61067800 | 0.83558300 | -0.02170800 |
| C | -2.86538900 | -0.52544400 | 0.06758000 |
| H | -2.09102600 | -1.26071500 | 0.26859400 |
| C | -4.20843000 | -0.86602800 | -0.10985200 |
| C | -5.02543500 | 0.22566200 | -0.34425200 |
| H | -4.56597700 | -1.89037600 | -0.07337200 |
| S | -4.09646100 | 1.70068800 | -0.33432500 |
| S | 4.10776200 | 1.80892700 | -0.01226600 |
| C | 2.91159100 | -0.44530500 | 0.33083700 |
| H | 2.14603300 | -1.19533100 | 0.51012100 |
| C | 6.52924800 | 0.34935400 | -0.06186500 |
| C | 7.31357600 | 1.52437200 | -0.10028200 |
| C | 7.21590200 | -0.87042400 | -0.13270900 |
| C | 8.69284100 | 1.45232500 | -0.19258700 |
| C | 8.59844600 | -0.94378400 | -0.22140100 |
| H | 6.64208800 | -1.79020700 | -0.12860300 |
| C | 9.38110000 | 0.23038900 | -0.25860000 |
| H | 9.26526200 | 2.37279200 | -0.19219600 |
| C | -6.47562400 | 0.18530500 | -0.49107200 |
| C | -7.23783500 | 1.27410000 | -0.97086400 |
| C | -7.17593600 | -0.97648100 | -0.14217200 |
| C | -8.61221400 | 1.16832700 | -1.10022500 |
| C | -8.55373000 | -1.08257600 | -0.26960700 |
| H | -6.61929900 | -1.81118400 | 0.26940300 |
| C | -9.31304900 | -0.00135100 | -0.76600800 |
| H | -9.17460700 | 2.02700400 | -1.44900000 |
| O | 6.63640100 | 2.69441200 | -0.03182000 |
| O | -6.54596400 | 2.39754700 | -1.27549900 |
| O | -9.24750800 | -2.19151400 | 0.07081000 |
| O | 9.27483400 | -2.11245400 | -0.28402000 |
| C | -7.24393000 | 3.53370000 | -1.72365600 |
| H | -7.76828400 | 3.33730000 | -2.66930600 |
| H | -7.96808400 | 3.88413200 | -0.97510000 |
| H | -6.49270300 | 4.30841400 | -1.88462600 |
| C | 7.34908500 | 3.90704200 | -0.04444000 |
| H | 8.04035900 | 3.97625600 | 0.80685300 |
| H | 7.91260100 | 4.03317000 | -0.97931700 |
| H | 6.60199600 | 4.69851200 | 0.03498000 |
| C | -10.75743700 | -0.04510400 | -0.93187900 |
| C | -11.51335800 | 0.91056300 | -1.60827200 |
| S | -11.77335300 | -1.29236700 | -0.26482000 |
| C | -12.87561400 | 0.65358500 | -1.57356600 |
| H | -11.08144700 | 1.75943900 | -2.12706800 |
| C | -13.21753900 | -0.50587800 | -0.87357500 |
| H | -13.63171800 | 1.27785600 | -2.04339600 |
| C | 10.83172900 | 0.22383800 | -0.36289500 |
| C | 11.62478400 | 1.34875600 | -0.58491700 |
| S | 11.80970700 | -1.20628800 | -0.18044500 |
| C | 12.98239000 | 1.06712600 | -0.59118100 |
| H | 11.22526800 | 2.34305400 | -0.75281400 |
| C | 13.28518500 | -0.27965000 | -0.37702500 |
| H | 13.76294700 | 1.80725400 | -0.74996700 |
| C | 8.56393800 | -3.32685400 | -0.22821200 |
| H | 9.31201300 | -4.11886100 | -0.28373900 |
| H | 8.00482200 | -3.42045800 | 0.71267700 |
| H | 7.86937300 | -3.42393100 | -1.07365400 |
| C | -8.55909400 | -3.30834000 | 0.58264500 |
| H | -7.82761000 | -3.69355200 | -0.14092200 |
| H | -8.04586100 | -3.06801400 | 1.52362200 |
| H | -9.31620600 | -4.07092600 | 0.77049700 |
| C | -14.57658700 | -0.87816900 | -0.75477700 |
| H | -15.21000800 | -0.15130000 | -1.26148700 |
| C | 14.63498800 | -0.70043000 | -0.35660500 |
| H | 15.29625700 | 0.14975300 | -0.51914600 |
| C | -15.24002800 | -1.91864000 | -0.15047100 |
| C | -14.60375700 | -3.03401000 | 0.59290900 |
| C | -16.68881400 | -2.10271800 | -0.13620100 |
| C | -15.71206200 | -3.88983200 | 1.05091600 |
| C | 15.26152600 | -1.90983500 | -0.17364000 |
| C | 14.58172800 | -3.20555400 | 0.06846900 |
| C | 16.70638500 | -2.12472400 | -0.17742600 |
| C | 15.65883800 | -4.20018900 | 0.21174200 |
| C | -16.94891700 | -3.33377200 | 0.61367100 |
| C | -18.02951600 | -4.07764300 | 1.00244400 |
| C | -15.84078800 | -5.03960800 | 1.76197600 |
| H | -19.08579000 | -3.91612600 | 0.82577800 |
| C | 16.91982000 | -3.55361000 | 0.06579700 |
| C | 17.97370900 | -4.41709500 | 0.18875200 |
| C | 15.74336000 | -5.53617300 | 0.44191600 |
| H | 19.03850700 | -4.22795000 | 0.12844700 |
| O | -13.41288600 | -3.20918200 | 0.78426800 |
| O | 13.38177300 | -3.40821800 | 0.13664600 |
| C | -17.45993300 | -0.12201900 | -1.41230000 |
| C | -19.05113600 | -1.70196900 | -0.53983500 |
| C | -17.68524200 | -1.32458100 | -0.68457800 |
| N | -17.28038300 | 0.86296200 | -2.00807600 |
| N | -20.16140900 | -2.02864500 | -0.40969600 |
| C | 17.73416400 | -1.22571000 | -0.36293200 |
| C | 17.55339200 | 0.16541500 | -0.60382100 |
| C | 19.08786200 | -1.66654800 | -0.32036800 |
| N | 20.18733700 | -2.04857500 | -0.28100100 |
| N | 17.40965600 | 1.30458300 | -0.80133700 |
| S | 17.40338400 | -6.02365500 | 0.48354600 |
| H | 14.94705300 | -6.25511800 | 0.58790600 |
| H | -15.06989300 | -5.65746100 | 2.20508100 |
| S | -17.51372000 | -5.46024900 | 1.90561500 |

**Table S5:** Cartesian coordinates of **DTPD4**

| **Atom** | **X-axis** | **Y-axis** | **Z-axis** |
| --- | --- | --- | --- |
| C | 0.70251700 | 3.04922800 | 0.43192600 |
| C | -0.73989000 | 3.03240000 | 0.38346900 |
| C | -1.53667600 | 4.25187100 | 0.58968500 |
| C | -0.78412300 | 5.44383500 | 0.99307700 |
| C | 1.45272300 | 4.28835700 | 0.68581300 |
| C | 1.28235100 | 1.79940200 | 0.26268900 |
| C | -1.27732000 | 1.77050700 | 0.17555900 |
| C | 0.64643300 | 5.46122700 | 1.03701900 |
| C | 1.16961200 | 6.67170600 | 1.41871800 |
| C | -1.35822700 | 6.64123600 | 1.34178900 |
| S | -0.11880700 | 7.79065800 | 1.73675900 |
| S | 0.02448700 | 0.62004800 | -0.02673500 |
| C | 2.59083200 | 7.08431300 | 1.56860700 |
| H | 3.14893900 | 6.33185600 | 2.13387300 |
| H | 3.07766000 | 7.15370500 | 0.58891200 |
| H | 2.67806500 | 8.05330600 | 2.06966400 |
| C | -2.79471600 | 7.02196600 | 1.40490900 |
| H | -3.22596700 | 7.07278100 | 0.39868200 |
| H | -3.36901600 | 6.26362700 | 1.94533600 |
| H | -2.93199100 | 7.99297200 | 1.89070400 |
| O | 2.67678800 | 4.35216100 | 0.61064100 |
| O | -2.75436300 | 4.28331700 | 0.43516200 |
| C | 2.62758500 | 1.26701200 | 0.29106800 |
| C | 4.26919100 | -0.39147300 | 0.35605100 |
| C | 5.07211300 | 0.71343800 | 0.12955200 |
| H | 4.65002400 | -1.39937800 | 0.48689500 |
| C | -2.60951600 | 1.20804600 | 0.11380400 |
| C | -2.86670500 | -0.14954700 | 0.23540900 |
| H | -2.09663300 | -0.87792000 | 0.47413800 |
| C | -4.20558600 | -0.49405400 | 0.03459700 |
| C | -5.01415200 | 0.59098200 | -0.25243500 |
| H | -4.56681200 | -1.51638600 | 0.08748200 |
| S | -4.08565400 | 2.06591400 | -0.25738700 |
| S | 4.10741800 | 2.16575600 | 0.06600500 |
| C | 2.90999000 | -0.08284500 | 0.44239500 |
| H | 2.14458700 | -0.82869000 | 0.63872400 |
| C | 6.52281400 | 0.69601900 | -0.01496800 |
| C | 7.31342500 | 1.86682100 | -0.06158500 |
| C | 7.19950800 | -0.52757700 | -0.10674400 |
| C | 8.69017100 | 1.78688200 | -0.18004400 |
| C | 8.57961900 | -0.60911900 | -0.22187900 |
| H | 6.62013700 | -1.44385800 | -0.09959200 |
| C | 9.36924300 | 0.56052900 | -0.26524800 |
| H | 9.26792000 | 2.70403000 | -0.18644400 |
| C | -6.45872900 | 0.54004000 | -0.44189200 |
| C | -7.20427000 | 1.59070300 | -1.02212300 |
| C | -7.16693200 | -0.60045700 | -0.04440300 |
| C | -8.56933600 | 1.46099800 | -1.21401600 |
| C | -8.53633500 | -0.72859000 | -0.22937500 |
| H | -6.62492700 | -1.39870600 | 0.45064800 |
| C | -9.27645100 | 0.30614700 | -0.84094800 |
| H | -9.11960800 | 2.28709600 | -1.65030800 |
| O | 6.64367500 | 3.03982100 | 0.02592400 |
| O | -6.50534000 | 2.70021400 | -1.36014800 |
| O | -9.23708200 | -1.81716900 | 0.15664400 |
| O | 9.24663500 | -1.78091300 | -0.30650100 |
| C | -7.19038400 | 3.79779100 | -1.91274500 |
| H | -7.66356200 | 3.53737400 | -2.86980000 |
| H | -7.95563400 | 4.18267900 | -1.22440400 |
| H | -6.44012500 | 4.57140600 | -2.08265800 |
| C | 7.36152100 | 4.24923100 | -0.00027600 |
| H | 8.06915100 | 4.31572400 | 0.83770400 |
| H | 7.90734600 | 4.37310000 | -0.94589700 |
| H | 6.61948300 | 5.04390700 | 0.09330200 |
| C | -10.70639100 | 0.22626800 | -1.09159500 |
| C | -11.43242000 | 1.12024200 | -1.87840800 |
| S | -11.74116500 | -0.99866300 | -0.41511000 |
| C | -12.78760800 | 0.83258400 | -1.92184200 |
| H | -10.98101600 | 1.94388800 | -2.42072200 |
| C | -13.15482800 | -0.28965000 | -1.17360900 |
| H | -13.52033100 | 1.40556200 | -2.48479700 |
| C | 10.81741700 | 0.54288200 | -0.39283100 |
| C | 11.62018400 | 1.66419200 | -0.60419500 |
| S | 11.78137000 | -0.90112100 | -0.25676400 |
| C | 12.97342700 | 1.36764500 | -0.64056500 |
| H | 11.22952800 | 2.66648700 | -0.74250200 |
| C | 13.26448900 | 0.01227400 | -0.46068300 |
| H | 13.75940300 | 2.10249400 | -0.79709200 |
| C | 8.53239000 | -2.99350200 | -0.24343000 |
| H | 9.27703300 | -3.78708600 | -0.31919400 |
| H | 7.99315900 | -3.09109700 | 0.70847300 |
| H | 7.82067100 | -3.08321800 | -1.07519300 |
| C | -8.56481200 | -2.89201100 | 0.77110000 |
| H | -7.79602800 | -3.31261400 | 0.10878800 |
| H | -8.10063000 | -2.58619300 | 1.71834100 |
| H | -9.32396300 | -3.64962400 | 0.97020100 |
| C | -14.50682900 | -0.69733600 | -1.13350100 |
| H | -15.12067000 | -0.02802900 | -1.73506100 |
| C | 14.60760000 | -0.42519500 | -0.47406400 |
| H | 15.27753200 | 0.41895900 | -0.63177900 |
| C | -15.18483900 | -1.71961400 | -0.51076200 |
| C | -14.57566500 | -2.74619300 | 0.36906500 |
| C | -16.62143600 | -1.95903200 | -0.60004900 |
| C | -15.69192900 | -3.59576500 | 0.81368500 |
| C | 15.22197300 | -1.64755900 | -0.32728000 |
| C | 14.52550000 | -2.93573800 | -0.09687800 |
| C | 16.66148200 | -1.88287500 | -0.36379100 |
| C | 15.59026500 | -3.94736500 | 0.00735600 |
| C | -16.90835600 | -3.12882800 | 0.23650500 |
| C | -17.99862600 | -3.86766900 | 0.60283900 |
| C | -15.85996800 | -4.68016400 | 1.61293800 |
| H | -19.04013500 | -3.75362400 | 0.32738400 |
| C | 16.86029400 | -3.32003700 | -0.15101400 |
| C | 17.90921100 | -4.19200600 | -0.06540600 |
| C | 15.67821700 | -5.28704200 | 0.21169000 |
| H | 18.97390100 | -4.00777600 | -0.14438200 |
| O | -13.40060500 | -2.86755800 | 0.66590100 |
| O | 13.32521100 | -3.12333700 | -0.00969800 |
| C | -17.34197300 | -0.14212700 | -2.12429000 |
| C | -18.94987200 | -1.70123900 | -1.24440000 |
| C | -17.59128800 | -1.27869700 | -1.30455700 |
| N | -17.14291400 | 0.78887000 | -2.79574800 |
| N | -20.05418900 | -2.06554000 | -1.17967800 |
| C | 17.69975500 | -0.99635000 | -0.55315600 |
| C | 17.53495700 | 0.40150800 | -0.76356500 |
| C | 19.04698600 | -1.45783300 | -0.54696000 |
| N | 20.14047800 | -1.85865600 | -0.53737900 |
| N | 17.40367400 | 1.54618800 | -0.93610000 |
| S | 17.34407500 | -5.80565700 | 0.21305600 |
| S | -17.53801900 | -5.15337200 | 1.66968600 |
| Cl | 14.40909500 | -6.40801500 | 0.44634900 |
| Cl | -14.66974800 | -5.53709300 | 2.49086000 |

**Table S6:** Cartesian coordinates of **DTPD5**

| **Atom** | **X-axis** | **Y-axis** | **Z-axis** |
| --- | --- | --- | --- |
| C | 0.71652900 | 2.87601900 | 0.07363900 |
| C | -0.72849300 | 2.86896700 | 0.06586800 |
| C | -1.50794100 | 4.10460400 | 0.22634800 |
| C | -0.73533300 | 5.31403200 | 0.52592200 |
| C | 1.48237200 | 4.11900700 | 0.24425100 |
| C | 1.28317800 | 1.61456000 | -0.04986600 |
| C | -1.28148200 | 1.60187400 | -0.06248000 |
| C | 0.69490600 | 5.32094500 | 0.53469900 |
| C | 1.23666300 | 6.55036100 | 0.81642600 |
| C | -1.29260300 | 6.53805300 | 0.80086500 |
| S | -0.03540900 | 7.70093000 | 1.08409100 |
| S | 0.00754600 | 0.43309700 | -0.24802500 |
| C | 2.66390800 | 6.96099000 | 0.90241000 |
| H | 3.21616900 | 6.29655900 | 1.57443800 |
| H | 3.14839100 | 6.87618800 | -0.07674100 |
| H | 2.76443700 | 7.99182300 | 1.25595700 |
| C | -2.72503600 | 6.93405200 | 0.86839100 |
| H | -3.19389900 | 6.85245000 | -0.11867900 |
| H | -3.28068800 | 6.25828800 | 1.52614000 |
| H | -2.84132100 | 7.96097500 | 1.22844300 |
| O | 2.70612900 | 4.16787800 | 0.15056600 |
| O | -2.73075700 | 4.14222600 | 0.11715600 |
| C | 2.62333700 | 1.07039100 | -0.02454500 |
| C | 4.25201500 | -0.59994400 | 0.08957700 |
| C | 5.06636700 | 0.49279500 | -0.15274300 |
| H | 4.62303100 | -1.60763600 | 0.24760300 |
| C | -2.61608900 | 1.04447800 | -0.04899600 |
| C | -2.87655900 | -0.30557800 | 0.13632800 |
| H | -2.10008400 | -1.03313000 | 0.35669500 |
| C | -4.22982700 | -0.64103400 | 0.05514400 |
| C | -5.05215700 | 0.44268400 | -0.20068000 |
| H | -4.59290600 | -1.65160200 | 0.21287400 |
| S | -4.11335400 | 1.90867600 | -0.30715800 |
| S | 4.11460400 | 1.95026900 | -0.26277500 |
| C | 2.89484500 | -0.27784300 | 0.15795200 |
| H | 2.12323000 | -1.01377800 | 0.36707100 |
| C | 6.52018300 | 0.46248700 | -0.26909300 |
| C | 7.32193000 | 1.62351200 | -0.32004500 |
| C | 7.18916900 | -0.76867300 | -0.31349500 |
| C | 8.70293400 | 1.52878400 | -0.38822600 |
| C | 8.57076700 | -0.86462900 | -0.38142000 |
| H | 6.60012100 | -1.67874800 | -0.30385600 |
| C | 9.37033300 | 0.29588500 | -0.42348300 |
| H | 9.29394200 | 2.43811600 | -0.38852200 |
| C | -6.50436400 | 0.39879200 | -0.33382700 |
| C | -7.31431000 | 1.55239900 | -0.40877700 |
| C | -7.16269400 | -0.83882500 | -0.37138300 |
| C | -8.69343400 | 1.44470100 | -0.49786300 |
| C | -8.54251300 | -0.94731700 | -0.45715200 |
| H | -6.56672200 | -1.74409000 | -0.34218400 |
| C | -9.35006200 | 0.20646300 | -0.52898400 |
| H | -9.29368100 | 2.34801400 | -0.51404700 |
| O | 6.66283300 | 2.80585200 | -0.28353900 |
| O | -6.66619300 | 2.74132100 | -0.37308000 |
| O | -9.19397400 | -2.13428000 | -0.48315500 |
| O | 9.23129200 | -2.04761400 | -0.41392300 |
| C | -7.41124100 | 3.93271300 | -0.41632700 |
| H | -7.98933600 | 4.01256400 | -1.34763900 |
| H | -8.09601600 | 4.01069000 | 0.43966700 |
| H | -6.68554500 | 4.74657100 | -0.37149800 |
| C | 7.39745200 | 4.00456800 | -0.30447600 |
| H | 8.06985200 | 4.07942100 | 0.56151500 |
| H | 7.98675100 | 4.09928600 | -1.22726800 |
| H | 6.66376900 | 4.81124300 | -0.26071200 |
| C | -10.80238000 | 0.16491600 | -0.63684000 |
| C | -11.61348800 | 1.22965300 | -1.00117300 |
| S | -11.75693100 | -1.24144000 | -0.25025100 |
| C | -12.97363400 | 0.92643500 | -0.95269600 |
| H | -11.22702400 | 2.19382500 | -1.31563600 |
| C | -13.24640900 | -0.37501800 | -0.55035700 |
| H | -13.76446100 | 1.62676600 | -1.20916900 |
| C | 10.82342700 | 0.26590100 | -0.50102400 |
| C | 11.65592700 | 1.34172300 | -0.76662600 |
| S | 11.77473800 | -1.16656400 | -0.20122500 |
| C | 13.01965700 | 1.03321000 | -0.71235400 |
| H | 11.28067900 | 2.33021800 | -1.01092200 |
| C | 13.27085900 | -0.29163400 | -0.40442100 |
| H | 13.81674100 | 1.74844400 | -0.89502100 |
| C | 8.49348900 | -3.24511400 | -0.35066000 |
| H | 9.22285700 | -4.05627300 | -0.37190900 |
| H | 7.90901100 | -3.30761500 | 0.57746000 |
| H | 7.81709600 | -3.34480400 | -1.21051000 |
| C | -8.45130000 | -3.32481600 | -0.37995200 |
| H | -7.76031600 | -3.44220800 | -1.22624700 |
| H | -7.88016600 | -3.36222200 | 0.55805300 |
| H | -9.17708800 | -4.13921900 | -0.39200900 |
| C | -14.59003200 | -0.84510300 | -0.45234200 |
| H | -15.30355700 | -0.06472000 | -0.73073700 |
| C | 14.56080300 | -0.88086700 | -0.27881600 |
| H | 15.40900000 | -0.21275300 | -0.44468900 |
| C | -15.15444900 | -2.02484800 | -0.08272300 |
| C | -14.48245700 | -3.24465600 | 0.35662700 |
| C | 14.90899100 | -2.15260500 | 0.02782500 |
| C | 16.32515500 | -2.53466900 | 0.11919500 |
| C | 15.27339300 | -4.59552200 | 0.63262700 |
| C | -16.75324900 | -3.91729100 | 0.52731600 |
| O | -13.28356000 | -3.44089000 | 0.43958400 |
| O | 17.28813300 | -1.82118000 | -0.07127600 |
| S | 15.10650600 | -6.18542900 | 1.01590100 |
| N | 16.43334500 | -3.89067600 | 0.47493800 |
| C | 17.75818600 | -4.49763600 | 0.60624900 |
| H | 18.41811900 | -3.70651400 | 0.97486400 |
| H | 17.67618700 | -5.28224200 | 1.36475400 |
| C | 18.24847200 | -5.04777600 | -0.71731400 |
| H | 17.56649000 | -5.81752400 | -1.09381600 |
| H | 18.32447300 | -4.24693900 | -1.46020000 |
| H | 19.23900600 | -5.49765300 | -0.59774800 |
| S | 13.87544200 | -3.53661100 | 0.36826000 |
| S | -16.90922600 | -2.26454400 | -0.06545200 |
| N | -15.42853000 | -4.22382800 | 0.69663600 |
| S | -18.04893200 | -4.89026800 | 0.81378000 |
| C | -14.96963200 | -5.53589400 | 1.15216000 |
| H | -15.72519700 | -5.91484900 | 1.84707000 |
| H | -14.03689900 | -5.35867800 | 1.69579500 |
| C | -14.75474800 | -6.48403200 | -0.01005800 |
| H | -15.68941400 | -6.64356900 | -0.55776500 |
| H | -14.00416000 | -6.08291200 | -0.69933500 |
| H | -14.40155300 | -7.45437300 | 0.35282600 |

**Table S7:** Cartesian coordinates of **DTPD6**

| **Atom** | **X-axis** | **Y-axis** | **Z-axis** |
| --- | --- | --- | --- |
| C | -0.86626300 | 3.54588500 | -0.35414800 |
| C | 0.57629900 | 3.52477200 | -0.30079900 |
| C | 1.36256500 | 4.75269900 | -0.11019100 |
| C | 0.59899200 | 6.00441600 | -0.11909200 |
| C | -1.62900900 | 4.79720500 | -0.22566900 |
| C | -1.43408400 | 2.30144300 | -0.58690600 |
| C | 1.12371100 | 2.26507600 | -0.49587100 |
| C | -0.83088700 | 6.02652600 | -0.17528800 |
| C | -1.36062900 | 7.29284200 | -0.15261400 |
| C | 1.16590800 | 7.25295600 | -0.05184100 |
| S | -0.07956000 | 8.46149300 | -0.07180200 |
| S | -0.16730500 | 1.09975100 | -0.67369300 |
| C | -2.78307200 | 7.72670700 | -0.18256700 |
| H | -3.31325900 | 7.24653500 | -1.01091700 |
| H | -3.30034600 | 7.41622300 | 0.73210100 |
| H | -2.86980700 | 8.81300300 | -0.28263600 |
| C | 2.60071400 | 7.63663700 | 0.02955100 |
| H | 3.05055000 | 7.24468300 | 0.94819900 |
| H | 3.16481300 | 7.19344100 | -0.79746700 |
| H | 2.72875000 | 8.72311700 | 0.00705900 |
| O | -2.85510200 | 4.82630200 | -0.16307500 |
| O | 2.58028600 | 4.74668500 | 0.04838400 |
| C | -2.77484200 | 1.78920200 | -0.76980200 |
| C | -4.40024400 | 0.20445000 | -1.31310600 |
| C | -5.20096400 | 1.15576700 | -0.70748200 |
| H | -4.78391900 | -0.70988700 | -1.75510900 |
| C | 2.45927200 | 1.71706200 | -0.59167300 |
| C | 2.73936600 | 0.48600500 | -1.16532200 |
| H | 1.99208800 | -0.10806500 | -1.68417700 |
| C | 4.07455800 | 0.09461800 | -1.04238600 |
| C | 4.85841100 | 1.00997800 | -0.36129700 |
| H | 4.45252200 | -0.83107100 | -1.46425500 |
| S | 3.91244000 | 2.40605600 | 0.08612500 |
| S | -4.25542500 | 2.53641900 | -0.22236600 |
| C | -3.04835400 | 0.55690600 | -1.34441200 |
| H | -2.28336000 | -0.06424900 | -1.80207200 |
| C | -6.63820400 | 1.03681500 | -0.49468700 |
| C | -7.47500500 | 2.13007600 | -0.18116800 |
| C | -7.24536000 | -0.22062200 | -0.59735500 |
| C | -8.83779100 | 1.94856200 | -0.01497400 |
| C | -8.61044900 | -0.40433200 | -0.42903400 |
| H | -6.61680700 | -1.08302000 | -0.79201400 |
| C | -9.45040400 | 0.69176900 | -0.14036900 |
| H | -9.45316900 | 2.80584600 | 0.23424100 |
| C | 6.27968700 | 0.86006500 | -0.06932300 |
| C | 7.06635500 | 1.87068800 | 0.52688100 |
| C | 6.92889200 | -0.34228000 | -0.38017900 |
| C | 8.41127900 | 1.66152800 | 0.78265900 |
| C | 8.27641200 | -0.55143500 | -0.12703700 |
| H | 6.35099600 | -1.14456400 | -0.82482000 |
| C | 9.05892400 | 0.45490500 | 0.47775900 |
| H | 8.99001300 | 2.46638100 | 1.22148200 |
| O | -6.86282000 | 3.33180300 | -0.05692700 |
| O | 6.42806800 | 3.02954600 | 0.81512700 |
| O | 8.91679300 | -1.70602000 | -0.42373300 |
| O | -9.21164800 | -1.61377200 | -0.51605200 |
| C | 7.14037800 | 4.07743100 | 1.42507700 |
| H | 7.54616900 | 3.77394700 | 2.40029500 |
| H | 7.96147700 | 4.43194500 | 0.78660200 |
| H | 6.42372600 | 4.88734500 | 1.56988300 |
| C | -7.63463200 | 4.46266600 | 0.26362000 |
| H | -8.39766100 | 4.65881600 | -0.50275700 |
| H | -8.12562000 | 4.35142200 | 1.24071000 |
| H | -6.94104500 | 5.30403900 | 0.30372800 |
| C | 10.46932600 | 0.29388000 | 0.80157000 |
| C | 11.22970500 | 1.18736500 | 1.54608500 |
| S | 11.43957200 | -1.05738300 | 0.27734000 |
| C | 12.55995100 | 0.80165000 | 1.68134600 |
| H | 10.82757200 | 2.08934200 | 1.99461600 |
| C | 12.86464500 | -0.39886700 | 1.04996400 |
| H | 13.30613700 | 1.36780900 | 2.23286300 |
| C | -10.88934700 | 0.57355400 | 0.03455400 |
| C | -11.78882700 | 1.61537800 | 0.21489800 |
| S | -11.73381400 | -0.95258800 | 0.06083200 |
| C | -13.11395500 | 1.20395700 | 0.37818200 |
| H | -11.49256300 | 2.65881500 | 0.21731800 |
| C | -13.27153700 | -0.17038800 | 0.32350400 |
| H | -13.95069400 | 1.88076100 | 0.52650500 |
| C | -8.43319000 | -2.75551500 | -0.79758600 |
| H | -9.12228600 | -3.60150600 | -0.81234200 |
| H | -7.94172300 | -2.67004000 | -1.77606600 |
| H | -7.67329600 | -2.92164700 | -0.02241600 |
| C | 8.20824600 | -2.74860800 | -1.05080000 |
| H | 7.38789900 | -3.11401500 | -0.41818000 |
| H | 7.80357100 | -2.43109300 | -2.02153200 |
| H | 8.92813200 | -3.55338700 | -1.20661000 |
| C | 14.18097200 | -0.93644200 | 1.10691100 |
| H | 14.84955400 | -0.28624700 | 1.67774900 |
| C | -14.50183400 | -0.86978300 | 0.44608700 |
| H | -15.39033000 | -0.25891800 | 0.62068000 |
| C | 14.76791300 | -2.05990100 | 0.61332400 |
| C | 14.16806400 | -3.12041100 | -0.18122200 |
| C | -14.75233200 | -2.19985500 | 0.37905800 |
| C | -16.11102200 | -2.71908400 | 0.53270700 |
| C | -14.89463500 | -4.70291700 | 0.20880500 |
| C | 16.38068400 | -3.90231200 | 0.01050800 |
| O | 13.01346600 | -3.21126800 | -0.55103700 |
| O | -17.12094800 | -2.08280600 | 0.74162200 |
| N | -16.10995400 | -4.12711200 | 0.39589400 |
| C | -17.39229400 | -4.83277200 | 0.53655400 |
| H | -18.14728200 | -4.10994300 | 0.21407600 |
| H | -17.40370600 | -5.67384500 | -0.16030400 |
| C | -17.63930200 | -5.26811300 | 1.96575100 |
| H | -16.87153900 | -5.96826200 | 2.31108800 |
| H | -17.65412500 | -4.39688900 | 2.62898100 |
| H | -18.60637200 | -5.77444800 | 2.03925600 |
| S | -13.60148900 | -3.50697400 | 0.11426300 |
| S | 16.48007700 | -2.40013300 | 0.91133700 |
| N | 15.13558300 | -4.09726400 | -0.50358000 |
| C | 14.70396300 | -5.25403500 | -1.30091100 |
| H | 15.51969800 | -5.53771500 | -1.96901300 |
| H | 13.87542200 | -4.88434800 | -1.91143400 |
| C | 14.25413400 | -6.40452500 | -0.42487800 |
| H | 15.06350300 | -6.75561600 | 0.22311800 |
| H | 13.40604600 | -6.09839300 | 0.19682200 |
| H | 13.94087400 | -7.24754300 | -1.04801000 |
| C | -14.57230600 | -6.04299100 | 0.09160200 |
| C | -15.49410900 | -7.11949500 | 0.19171500 |
| N | -16.22962300 | -8.01879600 | 0.28129200 |
| C | -13.20559600 | -6.37935600 | -0.12419900 |
| N | -12.07651300 | -6.60507500 | -0.30124500 |
| C | 17.49999600 | -4.71021700 | -0.09479300 |
| C | 17.54479100 | -5.96882000 | -0.75195400 |
| N | 17.60241100 | -7.00841200 | -1.27544900 |
| C | 18.70162700 | -4.26340900 | 0.52370100 |
| N | 19.66397600 | -3.86170200 | 1.04295800 |

**Table S8:** Calculated energies (*E*) and energy gap (*∆E*) for **DTPR** and **DTPD1**-**DTPD6** in *eV*.

| **Compounds** | **HOMO-1** | **LUMO+1** | ***ΔE*** | **HOMO-2** | **LUMO+2** | ***ΔE*** |
| --- | --- | --- | --- | --- | --- | --- |
| **DTPR** | -5.607 | -2.472 | 3.135 | -6.122 | -2.048 | 4.074 |
| **DTPD1** | -5.805 | -2.969 | 2.836 | -6.303 | -2.404 | 3.899 |
| **DTPD2** | -6.173 | -3.587 | 2.586 | -6.663 | -3.223 | 3,44 |
| **DTPD3** | -5.837 | -3.033 | 2.804 | -6.328 | -2.338 | 3.99 |
| **DTPD4** | -5.782 | -2.752 | 3.03 | -6.238 | -2.234 | 4.004 |
| **DTPD5** | -5.580 | -2.478 | 3.102 | -6.025 | -2.077 | 3.948 |
| **DTPD6** | -5.782 | -2.752 | 3.03 | -6.238 | -2.234 | 4.004 |

**Table S9:** Computed Open circuit voltage values of **DTPR** and **DTPD1- DTPD6.**

| **Compounds** | ***ΔE*** | ***V_oc_ (V)*** |
| --- | --- | --- |
| **DTPR** | 2.717 | 2.543 |
| **DTPD1** | 2.441 | 2.092 |
| **DTPD2** | 2.167 | 1.490 |
| **DTPD3** | 2.408 | 2.027 |
| **DTPD4** | 2.634 | 2.283 |
| **DTPD5**  **DTPD6** | 2.706  2.634 | 2.535  2.283 |

$\boldsymbol{\Delta}$***E*=** $E_{\mathrm{LUMO}}^{A}-E_{\mathrm{HOMO}}^{D}$

**Table S10:** Wave length, excitation energy and oscillator strength of **DTPR** and **DTPD1- DTPD6** in chloroform solvent.

| **Compounds** | **λ (*nm*)** | **E(*eV*)** | ***f*_os_** | **MO Contributions** |
| --- | --- | --- | --- | --- |
|  | 585.490 | 2.118 | 3.182 | H-1→L+1 (11%), H→L (80%), H→L+2 (2%), H→L+3 (3%) |
|  | 537.632 | 2.306 | 0.377 | H-1→L (18%), H→L+1 (73%), H-1→L+1 (2%), H→L+3 (2%) |
|  | 467.474 | 2.652 | 0.009 | H-1→L (71%), H→L+1 (16%), H-2→L+1 (5%), H→L+2 (3%) |
| **DTPR** | 459.351 | 2.699 | 0.180 | H-1→L+1 (44%), H→L+2 (11%), H→L+3 (36%), H-2→L (5%) |
|  | 456.056 | 2.719 | 0.058 | H→L+2 (60%), H→L+3 (24%), H-1→L+3 (3%), H→L+1 (6%) |
|  | 450.488 | 2.752 | 0.184 | H-1→L+1 (32%), H→L (16%), H→L+2 (16%), H→L+3 (24%), H-1→L+2 (4%), H-1→L+3 (2%) |
|  | 656.900 | 1.887 | 3.284 | H-1→L+1 (12%), H→L (79%), H→L+2 (4%) |
|  | 613.111 | 2.022 | 0.194 | H-1→L (16%), H→L+1 (80%) |
|  | 522.607 | 2.372 | 0.036 | H-1→L (73%), H→L+1 (18%), H-2→L+1 (5%) |
| **DTPD1** | 512.116 | 2.421 | 0.446 | H-1→L+1 (74%), H→L (17%), H-2→L (5%) |
|  | 498.626 | 2.487 | 0.228 | H→L+2 (73%), H-1→L+1 (4%), H-1→L+3 (7%), H→L (3%), H→L+4 (9%) |
|  | 480.891 | 2.578 | 0.146 | H-1→L+2 (11%), H→L+3 (69%), H→L+5 (12%) |
|  | 709.693 | 1.747 | 2.812 | H-1→L+1 (11%), H→L (80%), H→L+2 (4%), H→L+4 (2%) |
|  | 670.361 | 1.850 | 0.158 | H-1→L (12%), H→L+1 (85%) |
|  | 573.944 | 2.160 | 0.724 | H-1→L+3 (12%), H→L+2 (74%), H→L (7%), H→L+4 (3%) |
| **DTPD2** | 565.540 | 2.192 | 0.125 | H-1→L (60%), H→L+1 (13%), H→L+3 (14%), H-2→L+1 (4%), H-1→L+2 (6%) |
|  | 557.529 | 2.224 | 0.067 | H-1→L (17%), H-1→L+2 (10%), H→L+3 (64%) |
|  | 554.735 | 2.235 | 0.233 | H-1→L+1 (78%), H→L (11%), H-2→L (5%), H-1→L (2%) |
|  | 665.397 | 1.863 | 3.350 | H-1→L+1 (12%), H→L (80%), H→L+2 (5%) |
|  | 619.359 | 2.002 | 0.210 | H-1→L (15%), H→L+1 (81%) |
|  | 528.241 | 2.347 | 0.037 | H-1→L (75%), H→L+1 (17%), H-2→L+1 (5%) |
| **DTPD3** | 517.114 | 2.398 | 0.466 | H-1→L+1 (76%), H→L (16%), H-2→L (5%) |
|  | 485.505 | 2.554 | 0.186 | H→L+2 (86%), H-1→L+1 (3%), H-1→L+3 (2%), H→L (3%), H→L+5 (2%) |
|  | 467.421 | 2.653 | 0.160 | H→L+3 (88%), H-1→L+2 (3%), H→L+4 (3%) |
|  | 675.217 | 1.836 | 3.229 | H-1→L+1 (12%), H→L (81%), H→L+2 (4%) |
|  | 629.805 | 1.969 | 0.235 | H-1→L (15%), H→L+1 (82%) |
|  | 536.399 | 2.311 | 0.052 | H-1→L (74%), H→L+1 (17%), H-2→L+1 (5%) |
| **DTPD4** | 525.375 | 2.360 | 0.449 | H-1→L+1 (75%), H→L (16%), H-2→L (5%) |
|  | 485.277 | 2.555 | 0.243 | H→L+2 (84%), H-1→L+1 (3%), H-1→L+3 (3%), H→L (3%), H→L+5 (3%) |
|  | 466.383 | 2.658 | 0.173 | H→L+3 (83%), H-1→L+2 (4%), H→L+4 (7%) |
|  | 598.462 | 2.072 | 3.153 | H-1→L+1 (10%), H→L (74%), H→L+1 (5%), H→L+2 (6%) |
|  | 548.114 | 2.262 | 0.528 | H-1→L (19%), H→L+1 (70%), H→L (4%), H→L+3 (3%) |
|  | 478.405 | 2.592 | 0.066 | H-1→L (71%), H→L+1 (15%), H-2→L+1 (3%), H→L (5%) |
| **DTPD5** | 469.687 | 2.640 | 0.070 | H-1→L+1 (36%), H→L+2 (52%), H-2→L (3%), H→L+3 (5%) |
|  | 463.992 | 2.672 | 0.055 | H→L+2 (11%), H→L+3 (74%), H-1→L+2 (5%), H→L (2%), H→L+1 (3%) |
|  | 459.045 | 2.701 | 0.220 | H-1→L+1 (41%), H→L (13%), H→L+2 (23%), H-1→L+3 (5%), H→L+1 (4%), H→L+3 (9%) |
|  | 606.899 | 2.043 | 3.064 | H-1→L+1 (12%), H→L (73%), H→L+1 (6%), H→L+2 (5%) |
|  | 559.845 | 2.215 | 0.676 | H-1→L (19%), H→L+1 (70%), H→L (4%), H→L+3 (2%) |
| **DTPD6** | 485.524 | 2.554 | 0.099 | H-1→L (71%), H→L+1 (16%), H-2→L+1 (4%), H→L (5%) |
|  | 473.165 | 2.620 | 0.127 | H-1→L+1 (67%), H→L+2 (18%), H-2→L (5%), H→L (3%) |
|  | 465.054 | 2.666 | 0.169 | H-1→L+1 (10%), H→L (12%), H→L+2 (69%), H-1→L+3 (4%) |
|  | 462.797 | 2.679 | 0.047 | H→L+3 (87%), H-1→L+2 (3%), H→L+1 (3%) |

**Table S11:** The structural representation of acceptor groups with their IUPAC names and abbreviations.

|  |  |  |
| --- | --- | --- |
| 2-(2-methyl-3-oxo-2,3-dihydro-1*H*-inden-1-ylidene)malononitrile (**MIM**) | methyl 6-cyano-1-(dicyanomethylene)-2-methyl-3-oxo-2,3-dihydro-1*H*-indene-5-carboxylate (**MMC**) | 2-(5-methyl-6-oxo-5,6-dihydro-4*H*-cyclopenta[*c*]thiophen-4-ylidene)malononitrile (**MTM**) |
|  |  |  |
| 2-(1-chloro-5-methyl-6-oxo-5,6-dihydro-4*H*-cyclopenta[*c*]thiophen-4-ylidene)malononitrile (**CTM**) | 3-ethyl-5-methyl-2-thioxothiazolidin-4-one (**EMT**) | (*Z*)-2-(3-ethyl-5-methyl-4-oxothiazolidin-2-ylidene)-2-isocyanoacetonitrile (**EMI**) |

**Table S12:** The structural representation designed compounds with their IUPAC names and abbreviations.

| **Compound** | **IUPAC name** |
| --- | --- |
| **DTPR** | 2,2'-((5,5'-((5,5'-(5,7-dimethyl-4,8-dioxo-4,8-dihydrobenzo[1,2-c:4,5-c']dithiophene-1,3-diyl)bis(thiophene-5,2-diyl))bis(2,5-dimethoxy-4,1-phenylene))bis(thiophene-5,2-diyl))bis(methanylylidene))bis(1H-indene-1,3(2H)-dione) |
| **DTPD1** | 2,2'-((2Z,2'Z)-((5,5'-((5,5'-(5,7-dimethyl-4,8-dioxo-4,8-dihydrobenzo[1,2-c:4,5-c']dithiophene-1,3-diyl)bis(thiophene-5,2-diyl))bis(2,5-dimethoxy-4,1-phenylene))bis(thiophene-5,2-diyl))bis(methanylylidene))bis(3-oxo-2,3-dihydro-1H-indene-2,1-diylidene))dimalononitrile |
| **DTPD2** | (2Z,2'Z)-dimethyl 2,2'-((5,5'-((5,5'-(5,7-dimethyl-4,8-dioxo-4,8-dihydrobenzo[1,2-c:4,5-c']dithiophene-1,3-diyl)bis(thiophene-5,2-diyl))bis(2,5-dimethoxy-4,1-phenylene))bis(thiophene-5,2-diyl))bis(methanylylidene))bis(6-cyano-1-(dicyanomethylene)-3-oxo-2,3-dihydro-1H-indene-5-carboxylate) |
| **DTPD3** | 2,2'-((5Z,5'Z)-5,5'-((5,5'-((5,5'-(5,7-dimethyl-4,8-dioxo-4,8-dihydrobenzo[1,2-c:4,5-c']dithiophene-1,3-diyl)bis(thiophene-5,2-diyl))bis(2,5-dimethoxy-4,1-phenylene))bis(thiophene-5,2-diyl))bis(methanylylidene))bis(6-oxo-5,6-dihydro-4H-cyclopenta[c]thiophene-5,4-diylidene))dimalononitrile |
| **DTPD4** | 2,2'-((5Z,5'Z)-5,5'-((5,5'-((5,5'-(5,7-dimethyl-4,8-dioxo-4,8-dihydrobenzo[1,2-c:4,5-c']dithiophene-1,3-diyl)bis(thiophene-5,2-diyl))bis(2,5-dimethoxy-4,1-phenylene))bis(thiophene-5,2-diyl))bis(methanylylidene))bis(1-chloro-6-oxo-5,6-dihydro-4H-cyclopenta[c]thiophene-5,4-diylidene))dimalononitrile |
| **DTPD5** | 1-(5-(4-(5-((E)-(3-ethyl-4-oxo-2-thioxothiazolidin-5-ylidene)methyl)thiophen-2-yl)-2,5-dimethoxyphenyl)thiophen-2-yl)-3-(5-(4-(5-((Z)-(3-ethyl-4-oxo-2-thioxothiazolidin-5-ylidene)methyl)thiophen-2-yl)-2,5-dimethoxyphenyl)thiophen-2-yl)-5,7-dimethylbenzo[1,2-c:4,5-c']dithiophene-4,8-dione |
| **DTPD6** | 2-((Z)-5-((5-(4-(5-(3-(5-(4-(5-((E)-((Z)-2-(cyano(isocyano)methylene)-3-ethyl-4-oxothiazolidin-5-ylidene)methyl)thiophen-2-yl)-2,5-dimethoxyphenyl)thiophen-2-yl)-5,7-dimethyl-4,8-dioxo-4,8-dihydrobenzo[1,2-c:4,5-c']dithiophen-1-yl)thiophen-2-yl)-2,5-dimethoxyphenyl)thiophen-2-yl)methylene)-3-ethyl-4-oxothiazolidin-2-ylidene)malononitrile |

| **DTPR**   | |
| --- | --- |
| **DTPD1** | **DTPD2**   |
| **DTPD3** | **DTPD4**   |
| **DTPD5**   | **DTPD6**   |

**Figure S1.** Molecular structures of **DTPR** and **DTPD1**-**DTPD6**.
